# A fluorescent calix[4]arene with naphthalene units at the upper rim exhibits long fluorescence emission lifetime without fluorescence quenching†

**DOI:** 10.1039/d1ra01743h

**Published:** 2021-03-22

**Authors:** Masaki Takahashi, Naoya Tsuji, Kohei Yazaki, Yoshihisa Sei, Makoto Obata

**Affiliations:** Interdisciplinary Graduate School of Medicine and Engineering, University of Yamanashi 4-4-37 Takeda Kofu 400-8510 Japan tmasaki@yamanashi.ac.jp; Laboratory for Chemistry and Life Science, Institute of Innovative Research, Tokyo Institute of Technology 4259 Nagatsuta, Midori-ku Yokohama 226-8503 Japan

## Abstract

We synthesised a new compound with four naphthyl groups in the upper rims of calix[4]arene (1). Compared to the monomer unit, compound 1 has redshifted absorption and fluorescence, together with high fluorescence quantum yield and long fluorescence lifetime, which is extremely rare because long fluorescence lifetime emission tends to reduce the quantum yield. Single-crystal X-ray analysis and quantum calculations in the S_1_ state revealed π–π through-space interactions between naphthalene rings.

## Abstract 2

Macrocyclic structure brings long fluorescence lifetime emission without fluorescence quenching and TD-DFT calculations revealed π–π interactions between the naphthalene rings.

## Graphical abstract

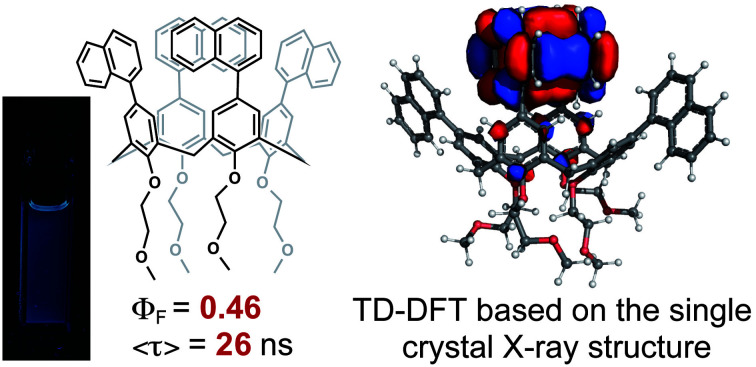

In 1954, Förster and Kasper first reported dimerised aromatic compounds in the excited state,^1^ which were termed ‘excimers’ by Stevens and Hutton.^2^ Excimers have attracted much attention in various fields such as organic solar cells,^3–5^ organic electronics,^6^ chemical sensors,^7^ and biotechnology^8–12^ because of their unique photophysical properties. In particular, with the development of time-resolved fluorescence imaging and stimulated emission depletion microscopy (STED) microscopes, there is a growing demand for fluorescent dyes with both high brightness and long fluorescence lifetime.^9–12^ Excimers with long emission lifetime are promising candidates for next-generation imaging probes.^13^

Many progresses have been made towards understanding the relationship between the molecular structure of organic dyes and their fluorescence intensity. However, there is little knowledge on the relationship between the fluorescence lifetime and molecular structure. Also, only a few fluorescent probes have achieved both high intensity and long lifetime.^13–15^ In recent years, it was reported that bright and long-lived fluorescence can be obtained from the excimer state^13,16–19^ that was conventionally thought to be the cause of quenching due to the low-energy excimer trap states with forbidden radiative transition and activated non-radiative process.^20–24^ However, most of these reports were in the solid state, where the molecular movement is suppressed and the luminescence occurs in a single crystal.^16–19^ In contrast, there are few reports of dyes with long fluorescent lifetime in solution systems with free molecular movement. The reported substances also suffer from low synthesis yields in the ring-forming reaction and difficulty in introducing functional groups (such as hydrophilic substituents).^13^ In general, the yield of the ring formation step is extremely low in the synthesis of cyclophanes.^13,25–27^ Therefore, there remains the need for new molecules that can be easily synthesised and chemically modified.

In this study, we adopted the calixarene skeleton as the macrocyclic structure. Calixarenes have been used in supramolecular chemistry,^28^ analytical chemistry,^29^ biochemistry,^30^ material science,^31^ and catalysts^32^ because of their easy molecular modification. Nevertheless, almost all studies introducing fluorescence sites do so at the lower rim of calixarene, while few reports considered incorporating fluorophores at the upper rim. Further, no researchers have investigated the fluorescence lifetime, and there was also no reported computational investigation of the excited state.^33–35^

Specifically, we synthesised a fluorescent molecule in which naphthyl group was introduced into the upper rim of calix[4]arene. The new molecule showed both a long fluorescence wavelength and a high quantum yield. After measuring the conformation of tetranaphthylcalix[4]arene in a single crystal, structural optimisation of the ground state and excited state (S_1_) was performed by time-dependent density functional theory (TD-DFT) at the DFT-D3-CAM-B3LYP/6-31G(d) level. The calculation takes into account effects such as dispersion force.^36–39^ Molecular orbital calculation confirmed that the π orbitals of the naphthalene rings of tetranaphthylcalixarene have a binding interactions in LUMO of the S_1_ state.

The synthesis of tetranaphthylcalix[4]arene (1) was carried out by deprotecting the *tert*-Bu groups of tetra-*tert*-butyl(tetrahydroxy)calix[4]arene, followed by introduction of substituents by Williamson ether synthesis of phenolic hydroxyl groups, and iodation by silver tetrafluoroacetate and iodine. The iodination was followed by Suzuki–Miyaura cross-coupling reaction with naphthylboronic acid pinacol ether (ESI†).

The target substance was characterised by ^1^H NMR, ^13^C NMR, HRMS of ESI-TOF-MS, and single-crystal X-ray diffraction (Fig. S7–S9 and Table S3, ESI†).

We also synthesised the phenylnaphthalene derivative 2 (Fig. 1), which is the unit molecule of 1. To compare its photophysical properties with that of 1, first we measured the absorption spectra. At 1 × 10^−4^ mol L^−1^ in chloroform solution, 1 and 2 have their maximum absorption wavelengths at 297 and 294 nm, and the molar absorption coefficients were 3.3 × 10^5^ and 1.0 × 10^5^ mol^−1^ L cm^−1^, respectively (Fig. 2). Their fluorescence spectra were measured in chloroform solution at 1 × 10^−4^ mol L^−1^ (Fig. 3). 1 has a broader fluorescence peak with maximum intensity at 389 nm, which is redshifted by 25 nm from that of 2. Furthermore, the fluorescence spectrum of the powder after grinding in a mortar was also measured and it was found that the fluorescence wavelength was increased by 22 nm (Fig. S14, ESI†). We also measured the absorption and fluorescence spectra at a lower concentration of 1 × 10^−5^ mol L^−1^ (Fig. S15, ESI†), and there was almost no change in the wavelength or shape of the peak. Therefore, the spectral changes in tetranaphthylcalix[4]arene from the unit model molecule are due to intramolecular rather than intermolecular interactions. Furthermore, the temperature dependence of fluorescence was investigated by measuring fluorescence by changing the temperature from 20 °C to 80 °C (Fig. S16†). As the temperature rose from 20 °C to 80 °C, a blue shift of the fluorescence wavelength of about 10 nm was observed, and the half-value width of the peak narrowed. These results indicate that as the temperature rises, the intramolecular interaction in the excited state weakens and the light emission becomes closer to that of 2.

**Fig. 1:**
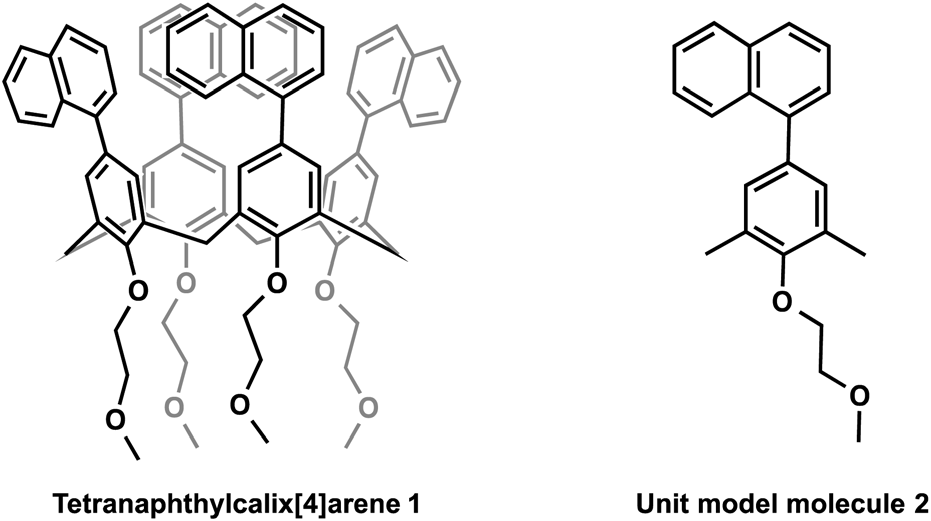
Molecular structures of tetranaphthylcalix[4]arene 1 and the unit model molecule 2.

**Fig. 2:**
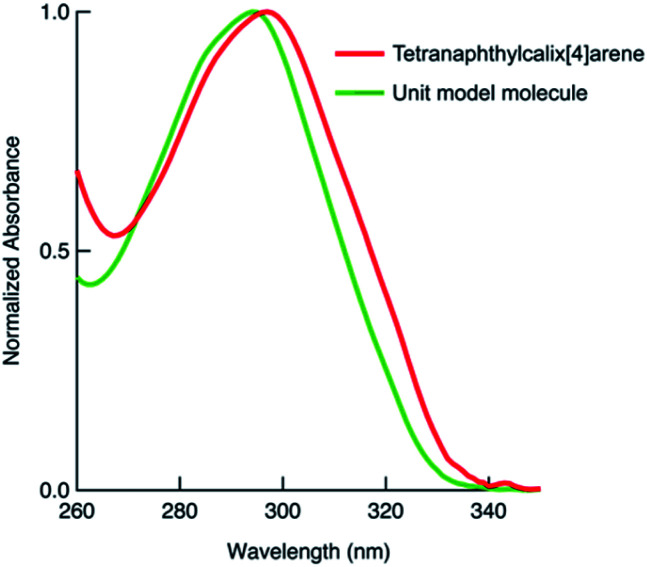
Absorption spectra of tetranaphthylcalix[4]arene 1 and the unit model molecule 2. Both spectra were measured in chloroform at 1 × 10^−4^ mol L^−1^, and normalised to the maximum absorption in the longer wavelength region.

**Fig. 3:**
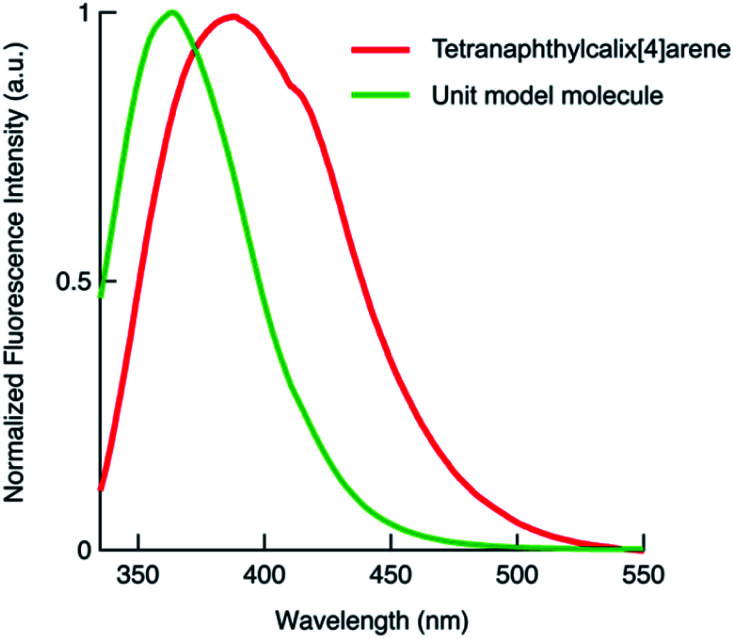
Fluorescence spectra of 1 and 2. Both spectra were measured in chloroform solution at 1 × 10^−4^ mol L^−1^, and normalised by the maximum intensity. 1: *λ*_ex_ = 310.5 nm, 2: *λ*_ex_ = 326.5 nm.

Single-crystal X-ray crystal structure analysis of 1 revealed that two of the four naphthyl groups facing each other had an intramolecular stacking structure, with a distance of 3.54 Å between them (Fig. 4 and Table S3, ESI†).

**Fig. 4:**
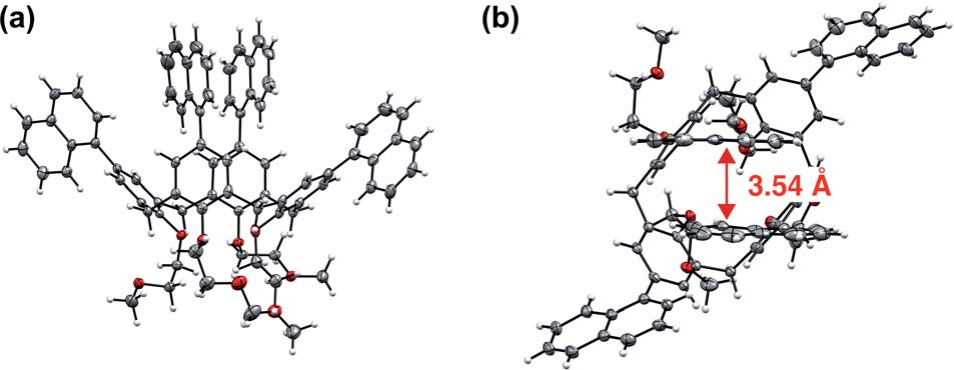
(a) Side and (b) top views of the structure of 1 determined by single-crystal X-ray crystallographic analysis. The structures were drawn by ORTEP program with the thermal ellipsoids set at 50% probability.

The macrocyclic structure improved the fluorescence quantum yield from 0.38 in 2 to 0.46 in 1 (Table 1). The unit model molecule showed single-exponential fluorescence decay with a fluorescence lifetime of 2.0 ns. In contrast, 1 displayed non-single exponential fluorescence decay. When the data were fitted using a two-component exponential equation (Fig. S17† and Table 1), the fitted weight ratio and fluorescence lifetime were *τ*_1_ = 2.4 ns, *A*_1_ = 0.95, *τ*_2_ = 48.0 ns, and *A*_2_ = 0.05. The area-weighted fluorescence lifetime 〈*τ*〉 was 26 ns. These lifetimes are very long compared to current commercial dyes with long fluorescence lifetimes for STED microscopes and time-gate imaging: Alexa Fluor 488 (4.1 ns),^11,40^ azadioxatriangulenium (ADOTA^+^) (25 ns),^14,41^ and SeTau425 NHS (26.2 ns).^15^ To investigate this characteristic property, the fluorescence emission rate constant *k*_f_ and the nonradiative decay rate constant *k*_nr_ were determined by the following equation:1
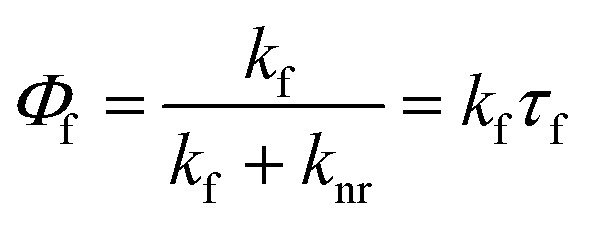
where *Φ*_f_ is the fluorescence quantum yield and *τ*_f_ is the fluorescence lifetime. The values are summarized in Table 1. *k*_nr_ decreased by more than one order of magnitude from 0.31 ns^−1^ to 0.021 ns^−1^ for 1. The small nonradiative decay rate of 1 is probably due to the suppression of molecular motion by the rigid macrocyclic structure of 1. On the other hand, the origin of the small *k*_f_ has been not identified. Furthermore, the ground and excited electronic states of 1 were obtained using density functional theory (DFT) and time-dependent (TD)-DFT calculations based on the structure from single-crystal X-ray diffraction. All calculations were performed at the DFT-D3-CAM-B3LYP/6-31 (d) level of theory (see Computational details in the ESI†). First, the structure of the ground state was optimised. The distance of the naphthalene rings facing each other, which is the focus of this study, was slightly smaller in the crystalline state (3.59 Å) compared to 3.49 Å of the ground state structure. Similar calculation was carried out for the ground state structure of 2. Then, the absorption spectra of both compounds were predicted by TD-DFT calculations. The absorption wavelength of 1 is longer, which qualitatively agrees with the experimental results (Fig. 2, S18, Tables S1 and S2, ESI†). Thus, it was possible to explain the change in absorption spectrum by calculating the molecular orbitals. In particular, for the first two excited states S_1_ and S_2_, the main constituent orbitals are HOMO to LUMO+2 and HOMO to LUMO+3 (Table S1, ESI†). These results indicate that the redshift of absorption in 1 involves the orbital of the intramolecular naphthalene units facing the molecule (Fig. S19, ESI†). Furthermore, structural optimisation of the S_1_ state was performed for compound 1 using the same level of theory and basis functions. The results further reduced the interplanar distance of naphthalene rings in the S_1_ state to 3.18 Å (Fig. 5a). In the frontier orbitals calculated for the S_1_ geometry, there were clear binding interactions between the naphthalene rings according to the LUMO (Fig. 5b and S20, ESI†) and a small oscillator strength value (0.0019) of the HOMO ← LUMO transition in agreement with the lower value of *k*_f_ compared with that of 2. An oscillator strength value of HOMO ← LUMO transition of 2 with the optimized structure in S_1_ state is 0.700. Therefore, the reason why 1 has both a high fluorescence quantum yield and a long fluorescence lifetime is that a decrease in *k*_nr_ due to suppression of molecular motion by a rigid macrocyclic structure contributes more significantly to the fluorescence enhancement than the decrease in *k*_f_ by intramolecular electronic interaction in the excited state. Subsequently, our group has been developing new imaging dyes using this molecular skeleton having fluorescence sites that have a long conjugation length and therefore can be excited by visible light.

**Table 1:** Fluorescence quantum yields, lifetimes, and kinetic constants of 1 and 2 at 390 nm in THF (*λ*_ex_ = 340 nm)

Cmpd	*Φ*_f_ a	*τ*_1_ b [ns]	*τ*_2_ b [ns]	〈*τ*〉^c^ [ns]	*k*_f_ [ns^−1^]	*k*_nr_ [ns^−1^]
1	0.46	2.4 (95%)	48.0 (5%)	26	0.018	0.021
2	0.38	2.0	—	—	0.19	0.31

a Absolute fluorescence quantum yields.

b The area-weighted ratios (*A*_1_ and *A*_2_) are shown in parentheses.

c The area-weighted mean fluorescence lifetime was calculated according to the function in ESI.

**Fig. 5:**
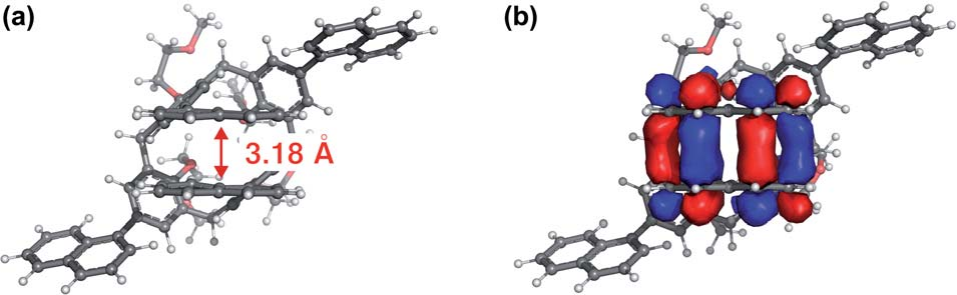
Top views of (a) the molecular structure of 1 in S_1_ excited state and with (b) superimposed LUMO. The S_1_ geometry was optimised by TD-DFT calculation.

In summary, we synthesised tetranaphthylcalix[4]arene (1). Such a structure with naphthalene substituents on the upper rim has never been reported before. A unit model molecule of 1 was also synthesised. For the optical properties, it was found that 1 has a higher fluorescence quantum yield and a fluorescence lifetime at least 11 times longer than that of the unit model molecule. These differences were interpreted based on single-crystal X-ray structure analysis and molecular orbital calculations, which revealed binding interactions between the naphthalene rings in the S_1_ state. These results will provide new insights into the molecular design of dyes with high fluorescence quantum yields and long fluorescence lifetimes.

## Conflicts of interest

There are no conflicts to declare.

## Supplementary Material

RA-011-D1RA01743H-s001

RA-011-D1RA01743H-s002
