# Supplementary material for: A fluorescent calix[4]arene with naphthalene units at the upper rim exhibits long fluorescence emission lifetime without fluorescence quenching
Source: RSC Adv. 2021 Mar 22;11(19):11651–4. doi: 10.1039/d1ra01743h (PMC8695987; doi:10.1039/d1ra01743h)
Supplement: RA-011-D1RA01743H-s001 [file RA-011-D1RA01743H-s001.pdf]

Electronic Supplementary Material (ESI) for RSC Advances.
This journal is © The Royal Society of Chemistry 2021

Electronic Supplementary Information

A Fluorescent Calix[4]arene with Naphthalene Units at the Upper Rim Exhibits Long Fluorescence Emission Lifetime without Fluorescence Quenching

Masaki Takahashi*, Naoya Tsuji, Kohei Yazaki, Yoshihisa Sei and Makoto Obata

Table of Contents:

1. Materials S2
2. Spectral Measurements S2
3. Computational Details S2
4. Syntheses, NMR Spectra, and ESI Spectra (Figures S1-S13) S3
5. Supplementary Figures and Tables (Figures S14-S20 and Tables S1-S6) S16
6. Supplementary References S38

1. Materials

All chemicals were purchased from Kanto Kagaku Co., Ltd., TCI Chemicals, or Aldrich, and used without further purification. Solvents for spectroscopic studies were of spectroscopic grade.

2. Spectral Measurements

^{1}H NMR and ^{13}C NMR spectra were measured on a Bruker Ascend 500 NMR spectrometer using tetramethylsilane (TMS) as the internal standard. X-ray crystallographic analysis was performed using a Bruker AXS D8 VENTURE/PHOTON 100 diffractometer. UV-vis spectra were measured using a JASCO V-570 spectrometer. Fluorescence spectra were measured using a JASCO FP-6500 fluorescence spectrometer. Fluorescence lifetime measurements were performed using a Hamamatsu Photonics model C11367 Quantaurus-Tau spectrometer. The fluorescence quantum yields were measured using a Hamamatsu Photonics C9920-02 absolute PL quantum yield measurement system at room temperature. ESI-TOF MS data were obtained using a Bruker micrOTOF II mass spectrometer in positive ion mode.

3. Computational Details

Gaussian16 was used for all geometry optimisations[1]. The structures of naphthylcalix[4]arene in the ground state and S_1 excited state, as well as the unit model in the ground state, were optimised with the long-range corrected CAM-B3LYP density functional[2] and the 6–31G* basis set[3]. In all calculations, dispersion interactions were accounted for by using the empirical dispersion correction D3 by Grimme et al.[4] All the minimum-energy structures were verified by frequency analyses.

4. Syntheses, NMR Spectra, and ESI Spectra

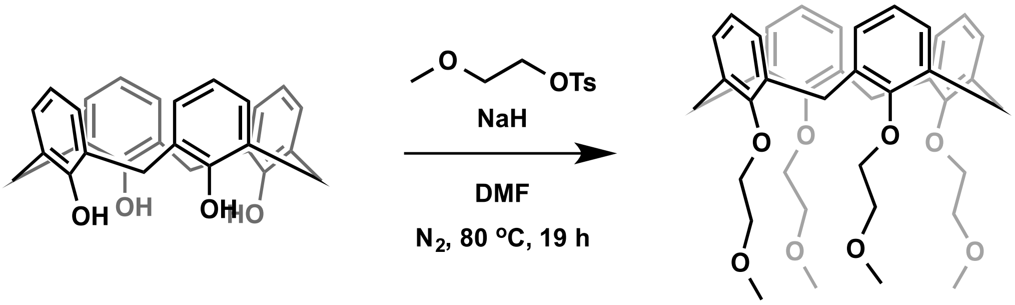

25,26,27,28-tetra(2’-methoxyethoxy)calix[4]arene

This compound was synthesised according to the literarture[5].Calix[4]arene (2.01 g, 4.71 mmol) and sodium hydride (0.678 g, 28.2 mmol) were added to a three-necked flask, and the atmosphere was replaced with nitrogen. 2-Methoxyethyl p-toluenesulfonate (7.22 g, 31.4 mmol) and N,N-dimethylformamide (24.0 mL) were added with a syringe, and the mixture was stirred at 80°C for 19 h. Then, potassium hydroxide aqueous solution (1.36 M, 20.0 mL) was added, and the mixture was stirred at 80°C for 23.5 h. The reaction system was transferred to a separatory funnel, water and ethyl acetate were added, and the organic and aqueous layers were separated. The aqueous layer was then extracted three times with ethyl acetate. The extracts were combined with the separated organic layer, washed with saturated brine, dried over anhydrous sodium sulfate, and the solvent was distilled off under reduced pressure using a rotary evaporator. Purification by silica gel chromatography (ethyl acetate:hexane = 1:2) gave a white powder (1.80 g, 2.75 mmol, 58%).

^{1}H NMR (500 MHz, $CDCl_3$): δ 6.64–6.56 (m, 12H), 4.48 (d, J = 5.4 Hz, 4H), 4.11 (t, J = 4.4 Hz, 8H), 3.83 (t, J = 4.4 Hz, 8H), 3.41 (s, 12H), 3.156 (d, J = 5.4 Hz, 4H). ^{13}C NMR (125.8 MHz, $CDCl_3$): δ 156.38, 135.12, 128.33, 122.39, 73.10, 71.97, 58.80, 30.87. ESI-TOF-MS *m/z* 679.3242 ($[M+Na]^+$, calcd: 679.3241).

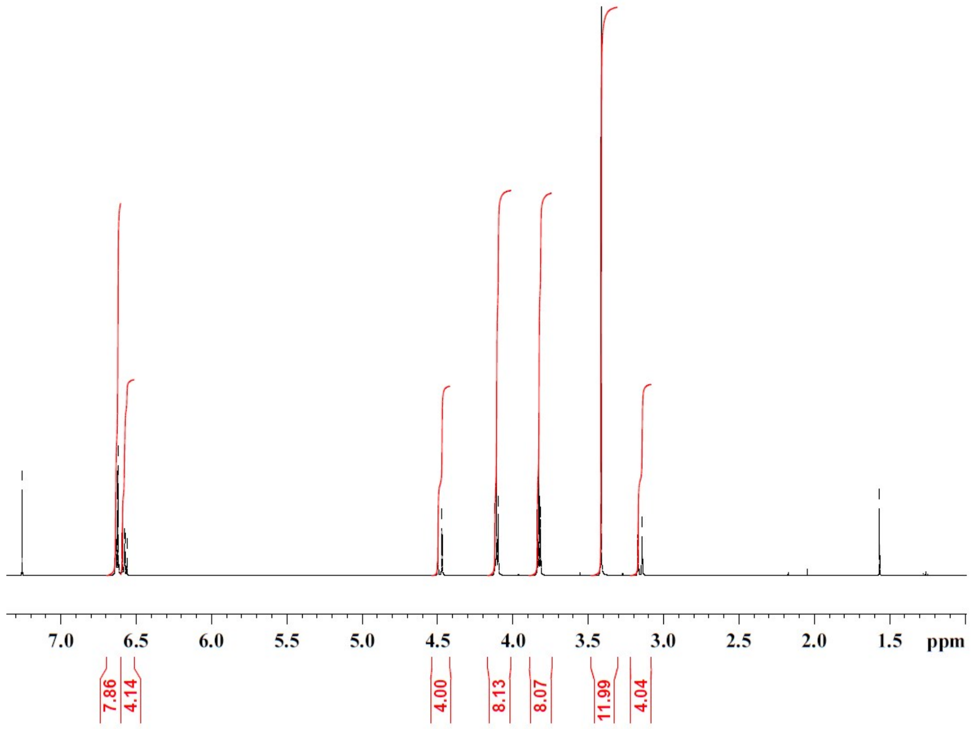

Figure S1. ^{1}H NMR spectrum of 25,26,27,28-tetra(2'-methoxyethoxy)calix[4]arene (500 MHz, $CDCl_3$, 298 K).

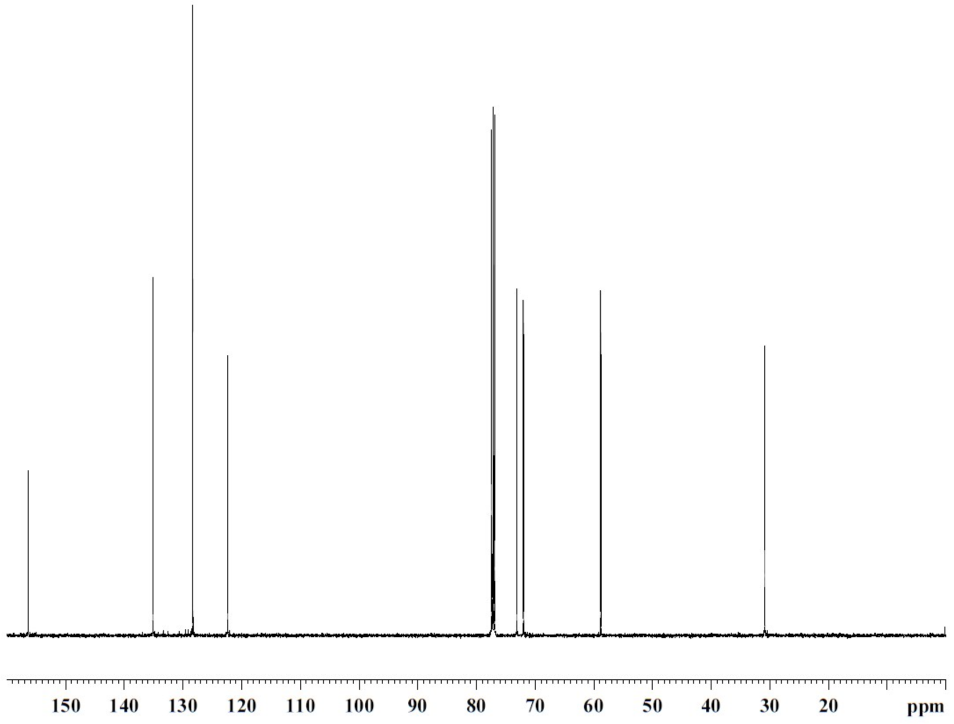

Figure S2. ^{13}C NMR spectrum of 25,26,27,28-tetra(2'-methoxyethoxy)calix[4]arene (125 MHz, $CDCl_3$, 298 K).

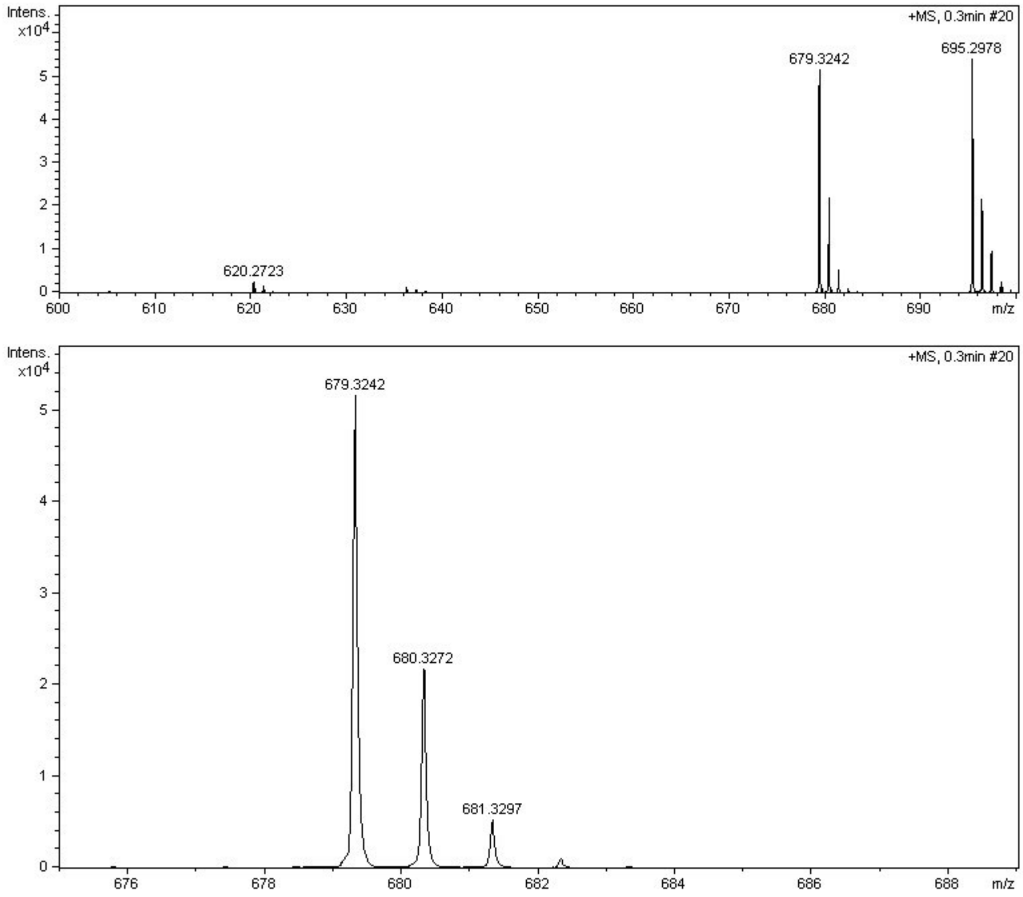

Figure S3. ESI-MS spectrum of 25,26,27,28-tetra(2'-methoxyethoxy)calix[4]arene.

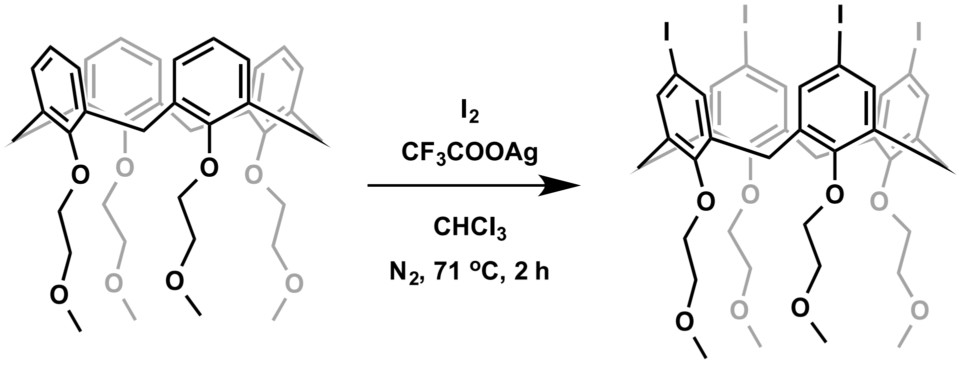

5,11,17,23-tetraiodo-25,26,27,28-tetra(2'-methoxyethoxy)calix[4]arene

This compound was synthesised according to the literarture[6]. 25,26,27,28-Tetra(2'-

methoxyethoxy)calix[4]arene (0.623 g, 0.949 mmol) and silver trifluoroacetate (1.36 g, 6.16 mmol) were added to a three-necked flask, and the atmosphere was replaced with nitrogen. Chloroform (32 mL) was added with a syringe, and the mixture was stirred at 71°C for 30 min, added with iodine (1.63 g, 6.42 mmol), and stirred at 71°C for 2 h. After filtration through Celite, the mixture was transferred to a separatory funnel, water and dichloromethane were added, and the organic and aqueous layers were separated. The aqueous layer was then extracted three times with dichloromethane. The organic layer was added with aqueous sodium sulfite solution (0.165 M, 75.0 mL), and the mixture was separated. The extracted organic layers were combined, washed with saturated brine, dried over anhydrous sodium sulfate, and the solvent was evaporated under reduced pressure using a rotary evaporator. Purification by silica gel chromatography (ethyl acetate:hexane = 1:1) gave a white powder (0.468 g, 0.403 mmol, 42%).

^{1}H NMR (500 MHz, $CDCl_3$): δ 7.01 (s, 8H), 4.37 (d, J = 5.4 Hz, 4H), 4.07 (t, J = 4.2 Hz, 8H), 3.74 (t, J = 4.2 Hz, 8H), 3.36 (s, 12H), 3.05 (d, J = 5.4 Hz, 4H). ^{13}C NMR (125.8 MHz, $CDCl_3$): δ 156.27, 137.26, 136.92, 86.65, 73.50, 71.79. 58.75, 30.25. ESI-TOF-MS *m/z* 1182.9119 ($[M+Na]^+$, calcd 1182.9107).

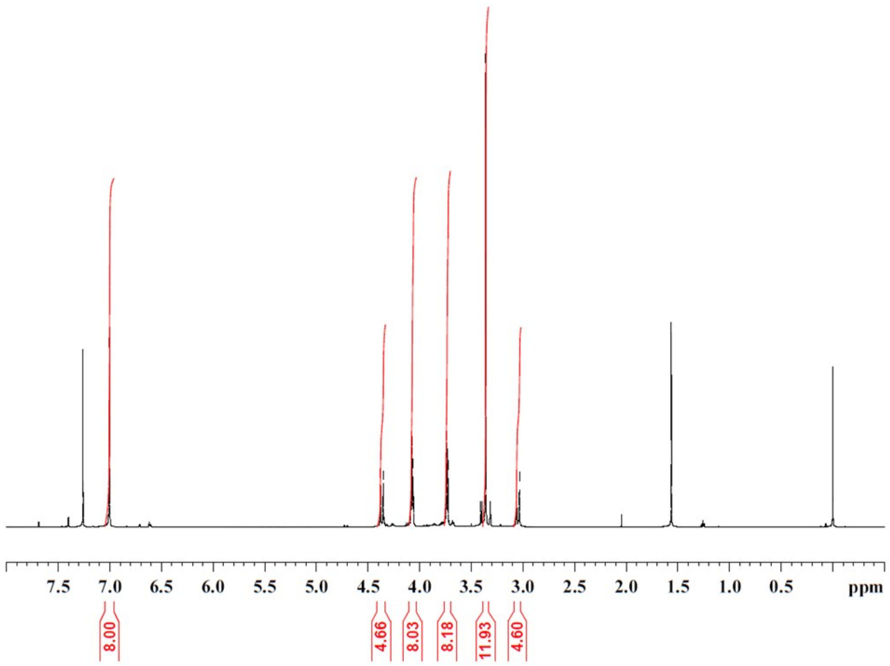

Figure S4. ^{1}H NMR spectrum of 5,11,17,23-tetraiodo-25,26,27,28-tetra(2'-methoxyethoxy)calix[4]arene (500 MHz, $CDCl_3$, 298 K).

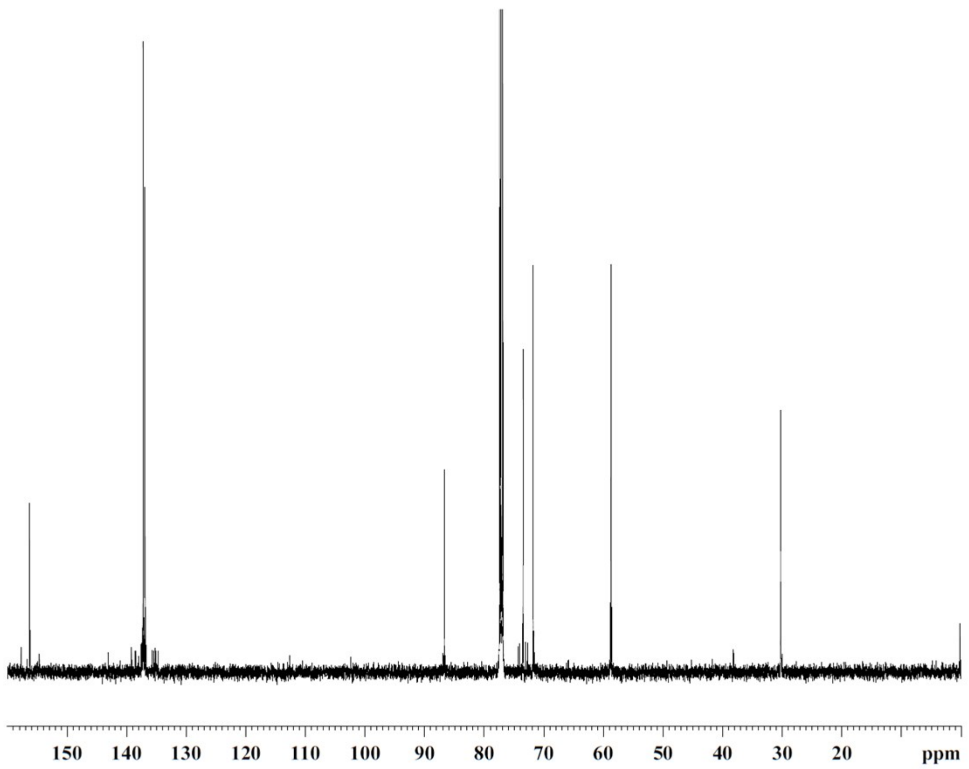

Figure S5. ^{13}C NMR spectrum of 5,11,17,23-tetraiodo-25,26,27,28-tetra(2'-methoxyethoxy)calix[4]arene (125 MHz, $CDCl_3$, 298 K).

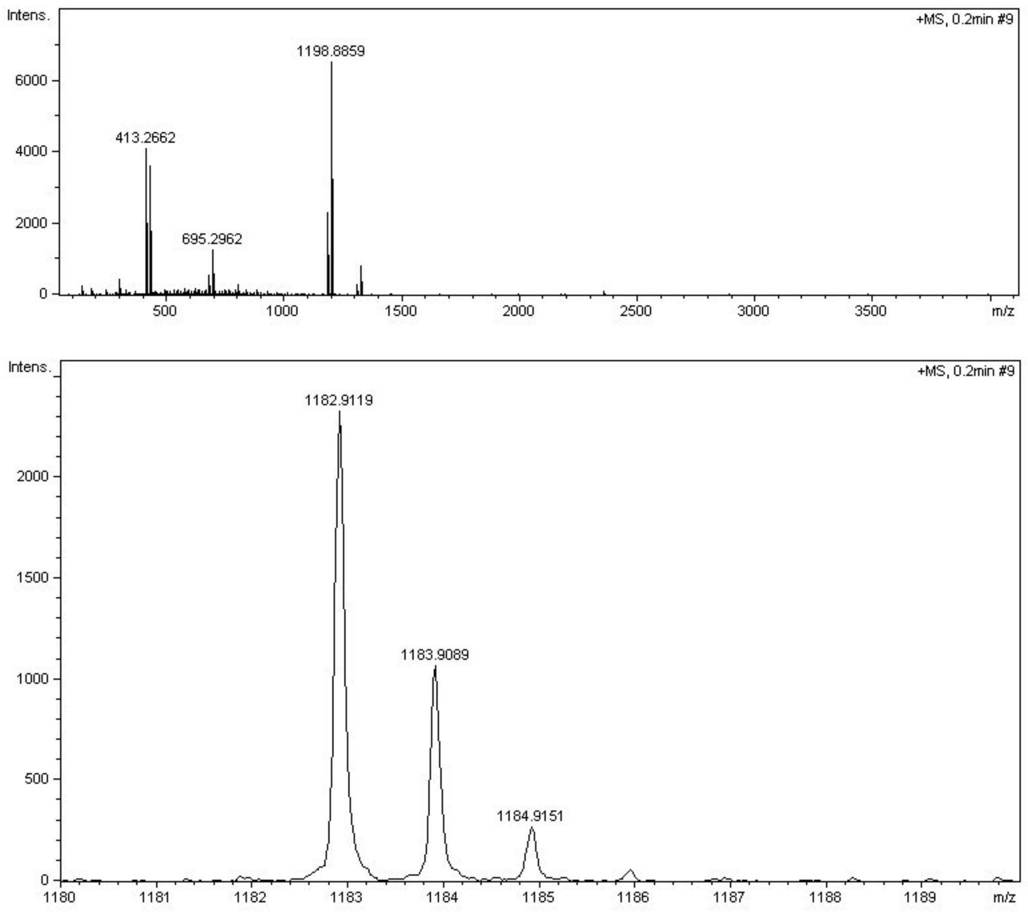

Figure S6. ESI-MS spectrum of 5,11,17,23-tetraiodo-25,26,27,28-tetra(2'-methoxyethoxy)calix[4]arene.

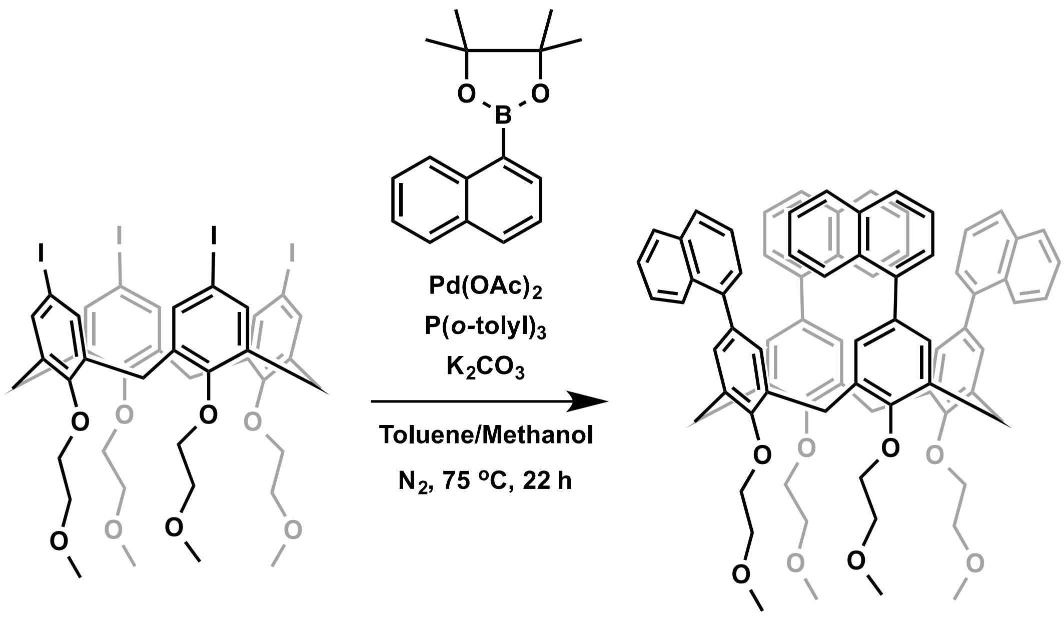

25,26,27,28-tetra(2'-methoxyethoxy)-5,11,17,23-tetranaphthylcalix[4]arene **1**

This compound was synthesised according to the literarture[7]. In a three-necked flask, 5,11,17,23-tetraiodo-25,26,27,28-tetra(2'-methoxyethoxy)calix[4]arene (0.337 g, 0.290 mmol), naphthalene-1-boronic acid pinacol ester (0.443 g, 1.47 mmol), palladium acetate (0.0137 g, 0.0609 mmol), tris(4-methylphenyl)phosphine (0.0360 g, 0.118 mmol), and potassium carbonate (0.642 g, 4.65 mmol) were added, and the atmosphere was replaced with nitrogen. Toluene (60.0 mL) and methanol (2.40 mL) were added with a syringe, and the mixture was stirred at 75°C for 22 h. The reaction system was transferred to a separatory funnel, water and ethyl acetate were added, and the organic and aqueous layers were separated. The aqueous layer was then extracted three times with ethyl acetate. The extract was combined with the separated organic layer, washed with saturated brine, dried over anhydrous sodium sulfate, and the solvent was distilled off under reduced pressure using a rotary evaporator. Purification by silica gel chromatography (ethyl acetate:hexane = 1:1) gave a white powder (0.206 g, 0.177 mmol, 61%).

^{1}H NMR (500 MHz, $CDCl_3$): δ 8.20–5.50 (br, 36H), 4.78 (d, J = 5.2 Hz, 4H) 4.38–4.05 (br, 16H), 3.54 (s, 12H), 3.37 (d, J = 5.2 Hz, 4H). ^{13}C NMR (125.8 MHz, $CDCl_3$) δ 139.65, 130.29, 126.85, 125.57, 71.94, 58.76, 31.11. Elemental analysis calcd for $C_{80}H_{72}O_8$ = C 82.73%; H 6.25%; O 11.02%, found = C 80.09%; H 6.19%; O 13.72%. ESI-TOF-MS *m/z* 1183.5235 ([M+Na]$^+$, calcd 1183.5119).

Single crystals of **1** for X-ray diffraction analysis were obtained by vapour diffusion of methanol into a 1,4-dioxane solution.

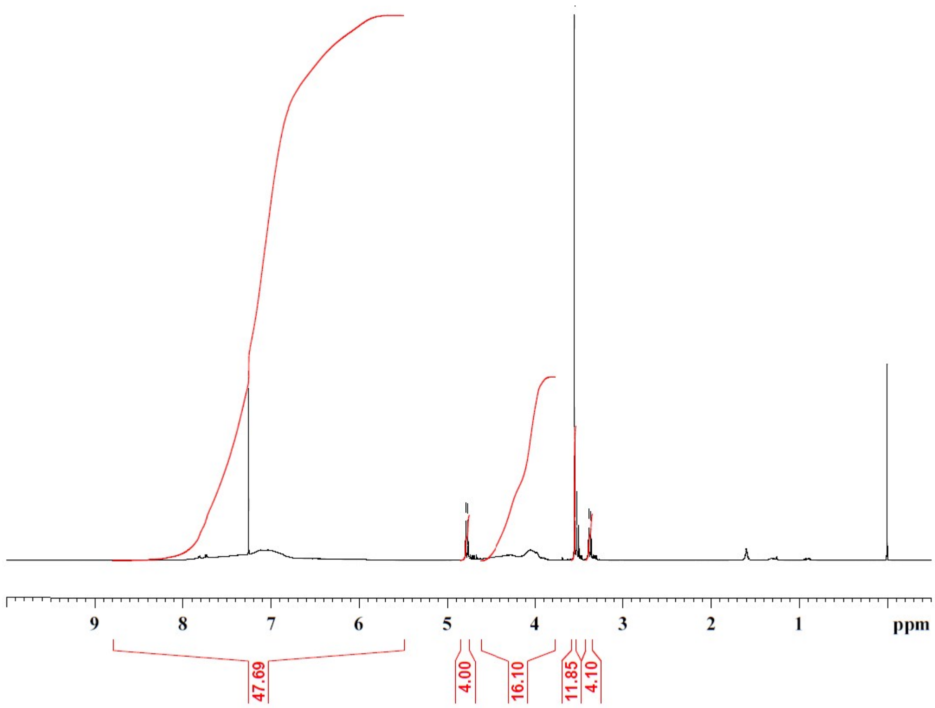

Figure S7. ^{1}H NMR spectrum of 25,26,27,28-tetra(2'-methoxyethoxy)-5,11,17,23-tetranaphthylcalix[4]arene **1** (500 MHz, $CDCl_3$, 298 K).

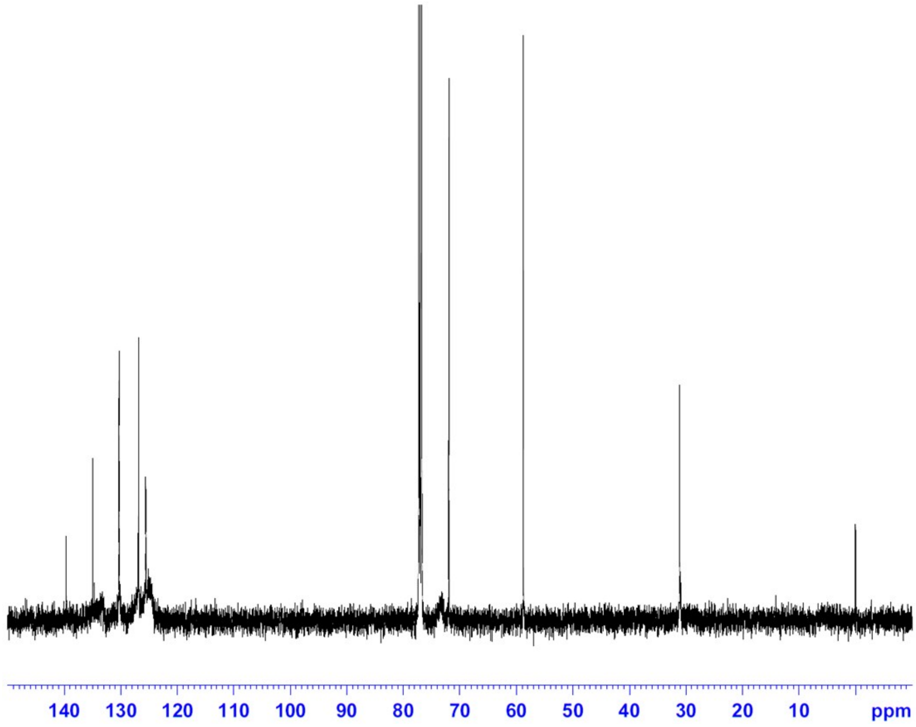

Figure S8. ^{13}C NMR spectrum of 25,26,27,28-tetra(2'-methoxyethoxy)-5,11,17,23-tetranaphthylcalix[4]arene **1** (125 MHz, $CDCl_3$, 298 K).

Display Report

Analysis Info

Analysis Name	D:\Data\Yamamoto\takahashi\20180207\000008.d	Acquisition Date	2/7/2018 3:50:47 PM
Method	esi_pos_wide.m	Operator	BDAL@DE
Sample Name	1_naphthylcalix	Instrument / Ser#	micrOTOF 10383
Comment	acetonitrle		

Acquisition Parameter

Source Type	ESI	Ion Polarity	Positive	Set Nebulizer	0.4 Bar
Focus	Not active			Set Dry Heater	180 °C
Scan Begin	50 m/z	Set Capillary	5000 V	Set Dry Gas	4.0 l/min
Scan End	4000 m/z	Set End Plate Offset	-500 V	Set Divert Valve	Waste

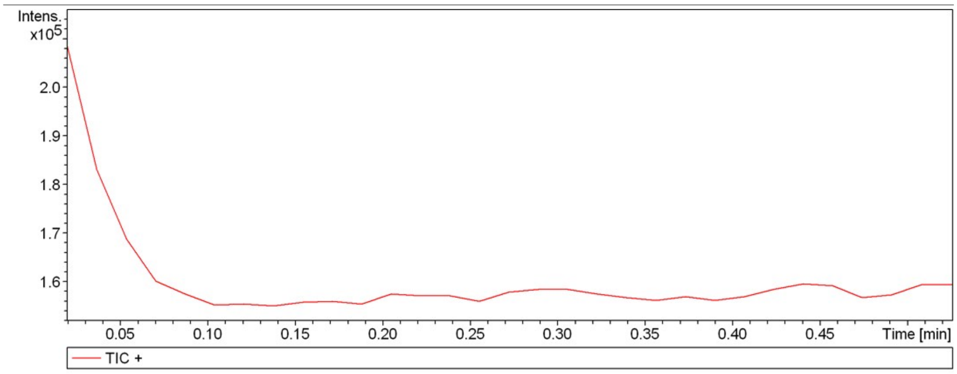

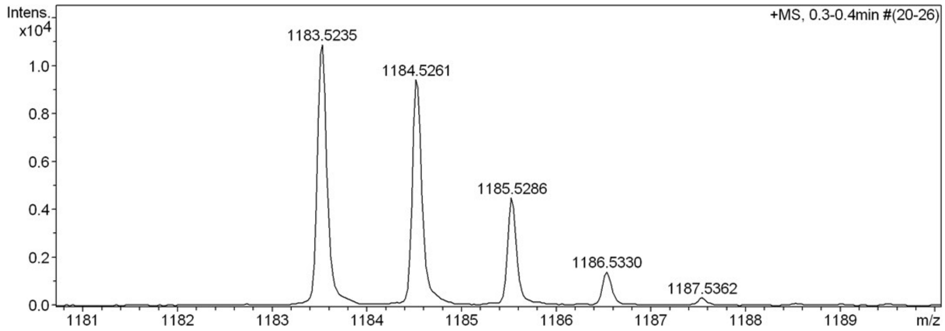

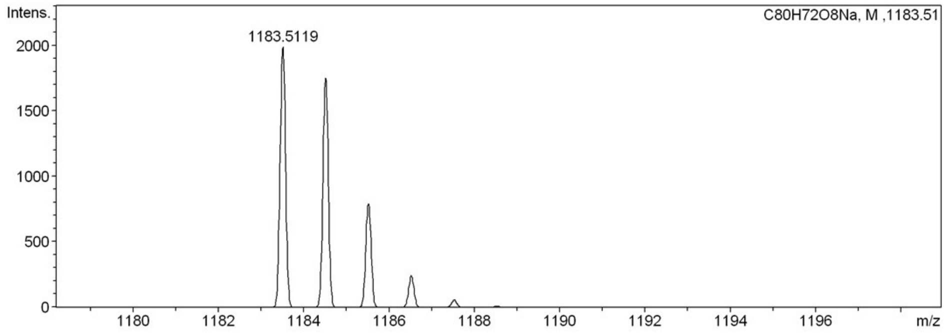

Bruker Compass DataAnalysis 4.0 printed: 2/7/2018 6:29:18 PM Page 1 of 1

Figure S9. ESI-MS spectrum of **1**.

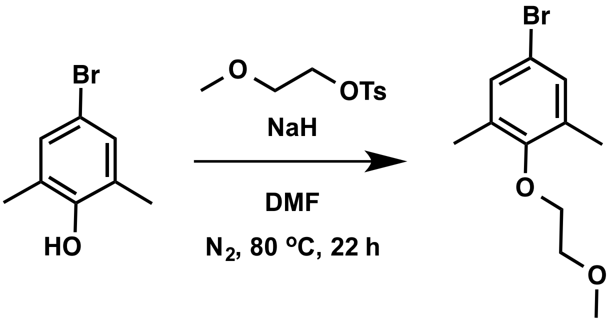

2-(2’-methoxyethoxy)-5-bromo-1,3-dimethylbenzene

4-Bromo-2,6-dimethylphenol (9.50 g, 47.2 mmol) and sodium hydride (0.937 g, 39.0 mmol) were added to a three-necked flask, and the atmosphere was replaced with nitrogen. 2-Methoxyethyl p-toluenesulfonate (16.3 g, 70.8 mmol) and N,N-dimethylformamide (100 mL) were added with a syringe, and the mixture was stirred at 80°C for 22 h. Then, potassium hydroxide aqueous solution (2.89 M, 20.0 mL) was added, and the mixture was stirred at 80°C for 24 h. The reaction system was transferred to a separatory funnel, water and ethyl acetate were added, and the organic and aqueous layers were separated. The aqueous layer was then extracted three times with ethyl acetate. The extract was combined with the separated organic layer, washed with saturated brine, dried over anhydrous sodium sulfate, and the solvent was distilled off under reduced pressure with a rotary evaporator to give a pale yellow liquid (10.4 g, 40.1 mmol, 85%).

^{1}H NMR (500 MHz, $CDCl_3$): δ 7.13 (s, 2H), 3.89 (t, J = 2.4 Hz, 2H), 3.71 (t, J = 3.8 Hz, 2H), 3.46 (s, 3H), 2.26 (s, 6H). ^{13}C NMR (125.8 MHz, $CDCl_3$): δ 155.03, 133.34, 131.51, 116.50, 71.96, 71.50, 59.34, 16.21.

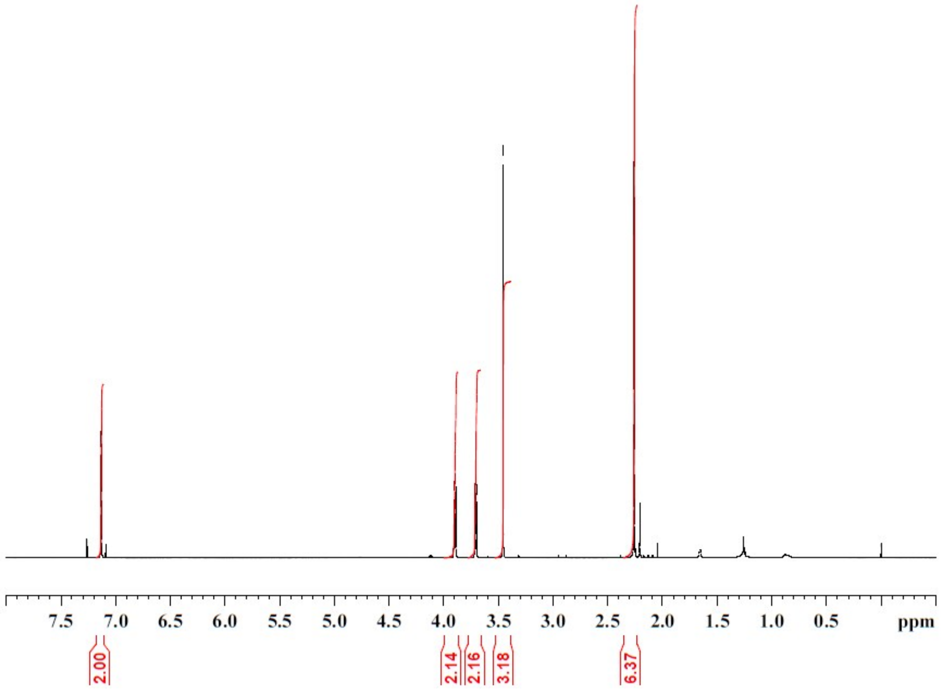

Figure S10. ^{1}H NMR spectrum of 2-(2'-methoxyethoxy)-5-bromo-1,3-dimethylbenzene (500 MHz, $CDCl_3$, 298 K).

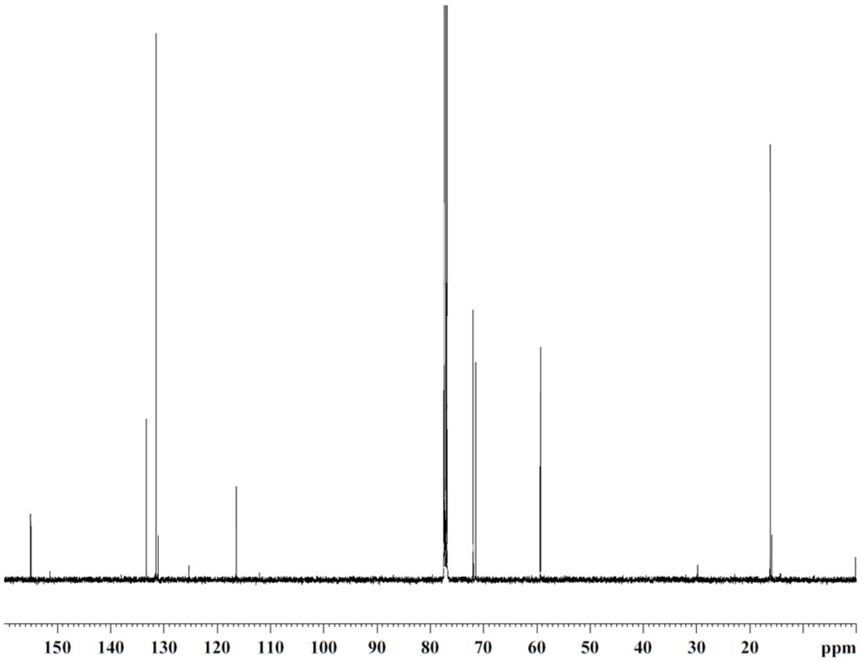

Figure S11. ^{13}C NMR spectrum of 2-(2'-methoxyethoxy)-5-bromo-1,3-dimethylbenzene (125 MHz, $CDCl_3$, 298 K).

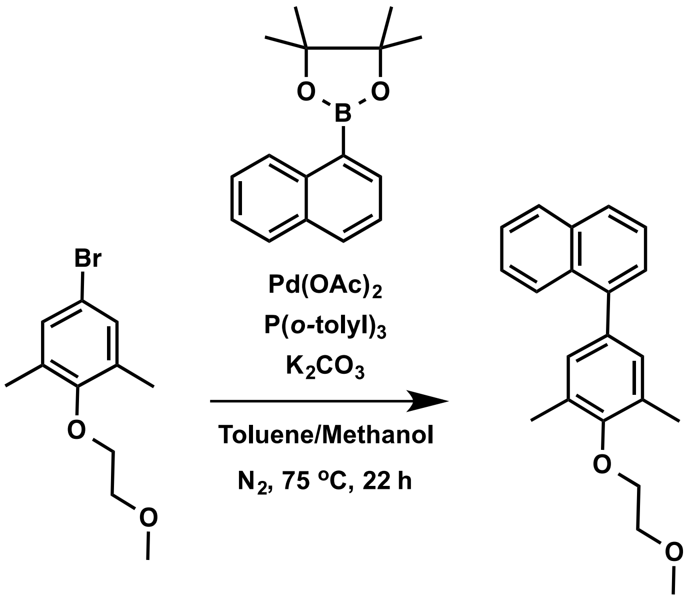

2-(2’-methoxyethoxy)-5-naphthyl-1,3-dimethylbenzene **2**

2-(2'-Methoxyethoxy)-5-bromo-1,3-dimethylbenzene (0.404 g, 1.56 mmol), naphthalene-1-boronic acid pinacol ester (0.675 g, 2.68 mmol), and palladium acetate (0.0246 g, 0.109 mmol) were added to a three-necked flask. tris(4-methylphenyl)phosphine (0.0674 g, 0.221 mmol), and potassium carbonate (1.23 g, 8.86 mmol) were added, and the atmosphere was replaced with nitrogen. Toluene (100 mL) and methanol (4.00 mL) were added with a syringe, and the mixture was stirred at 75°C for 17.5 h. The reaction system was transferred to a separatory funnel, water and ethyl acetate were added, and the organic and aqueous layers were separated. The aqueous layer was then extracted three times with ethyl acetate. The extract was combined with the separated organic layer, washed with saturated brine, dried over anhydrous sodium sulfate, and ethyl acetate was distilled off under reduced pressure using a rotary evaporator. Purification by silica gel chromatography (ethyl acetate:hexane = 1:7) gave a yellow liquid (0.280 g, 0.914 mmol, 59%).

^{1}H NMR (500 MHz, $CDCl_3$): δ 7.94 (d, J = 3.2 Hz, 1H), 7.88 (d, J = 3.2 Hz, 1H), 7.82 (d, J = 3.2 Hz, 1H), 7.50–7.38 (m, 4H), 7.13 (s, 2H), 4.04 (t, J = 3.8 Hz, 2H), 3.79 (t, J = 3.8 Hz, 2H), 2.37 (s, 6H). ^{13}C NMR (125.8 MHz, $CDCl_3$): δ 155.29, 140.20, 136.33, 133.91, 131.84, 130.80, 130.57, 128.34, 127.45, 126.91, 126.32, 125.99, 125.78, 125.47, 72.18, 71.54, 59.39, 16.51. Elemental analysis calcd for $C_{21}H_{22}O_2$ = C 82.32%; H 7.24%; O 10.44%, found = C 82.05%; H 6.92%; O 11.03%.

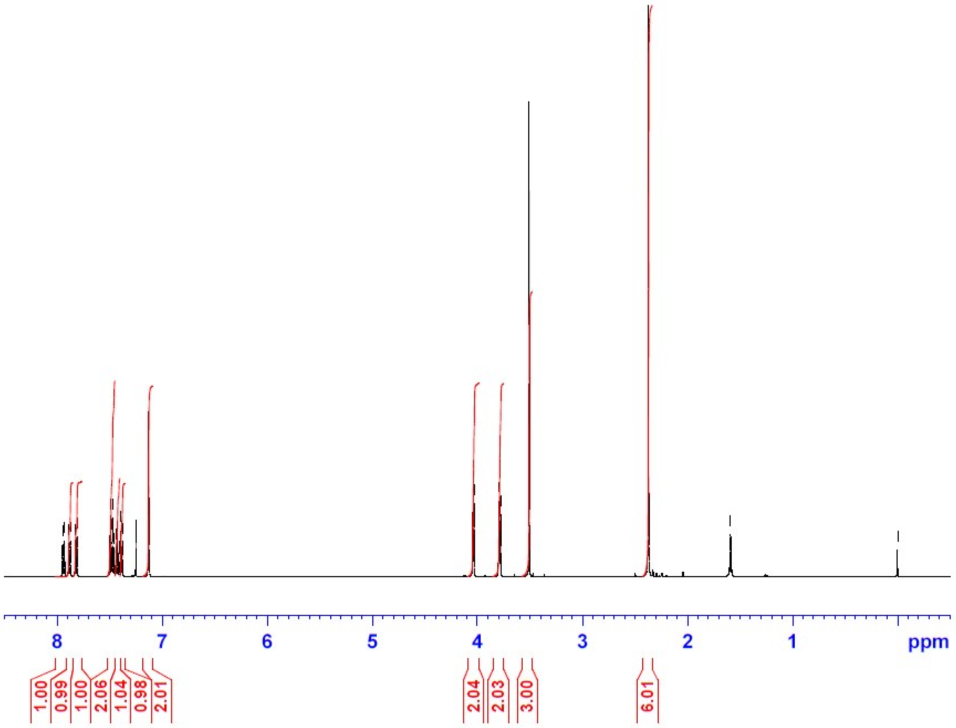

Figure S12. ^{1}H NMR spectrum of 2-(2'-methoxyethoxy)-5-naphthyl-1,3-dimethylbenzene **2** (500 MHz, $CDCl_3$, 298 K).

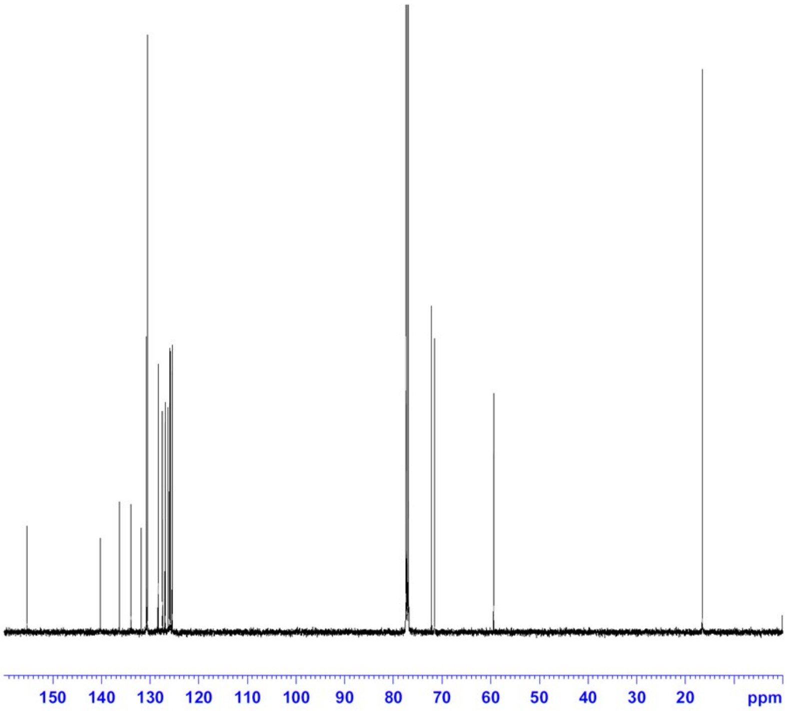

Figure S13. ^{13}C NMR spectrum of 2-(2'-methoxyethoxy)-5-naphthyl-1,3-dimethylbenzene **2** (125 MHz, $CDCl_3$, 298 K).

5. Supplementary Figures and Tables

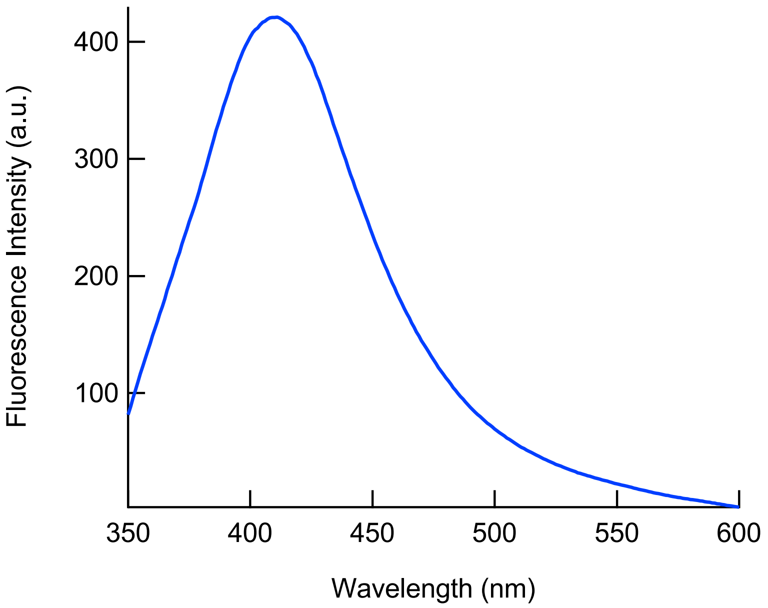

Figure S14. Fluorescence spectra of tetranaphthylcalix[4]arene **1** powder. λ_{ex} = 310.5 nm.

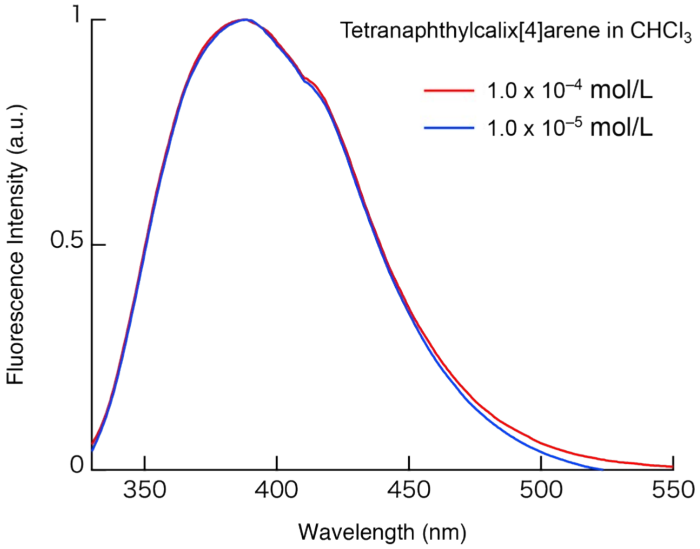

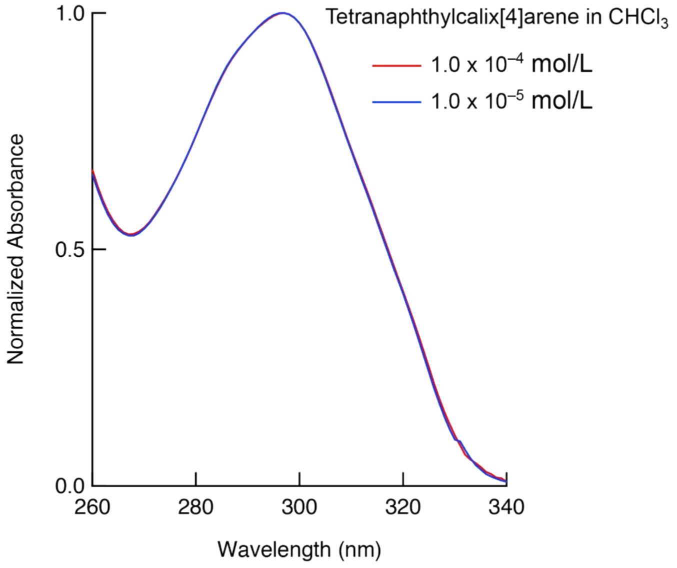

Figure S15. Fluorescence and UV-vis spectra of tetranaphthylcalix[4]arene **1** normalized at the maximum wavelength. In chloroform solution, 1×10^{-4} and 1×10^{-5} mol/L.

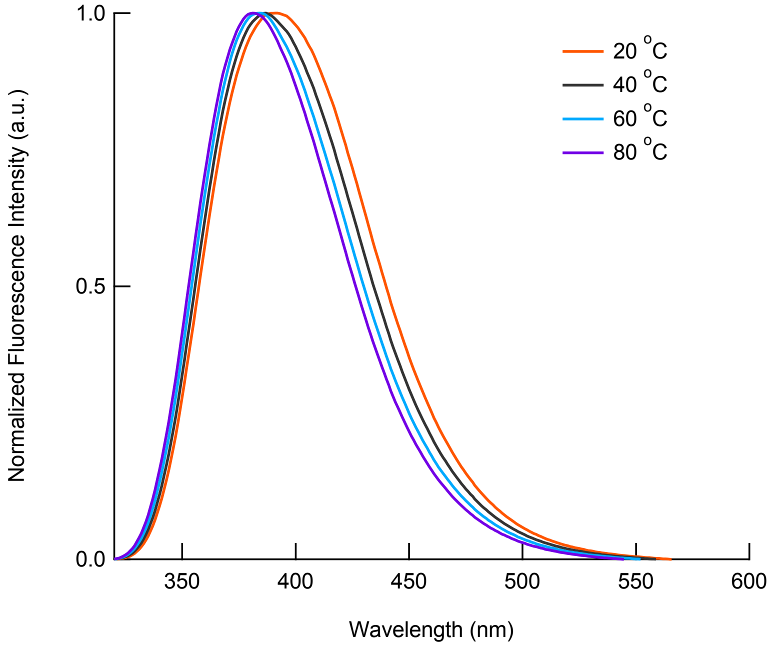

Figure S16. Fluorescence spectra of tetranaphthylcalix[4]arene **1** normalized at the maximum wavelength by changing the temperature from 20 °C to 80 °C. In dimethyl sulfoxide solution, 1×10^{-5} mol/L.

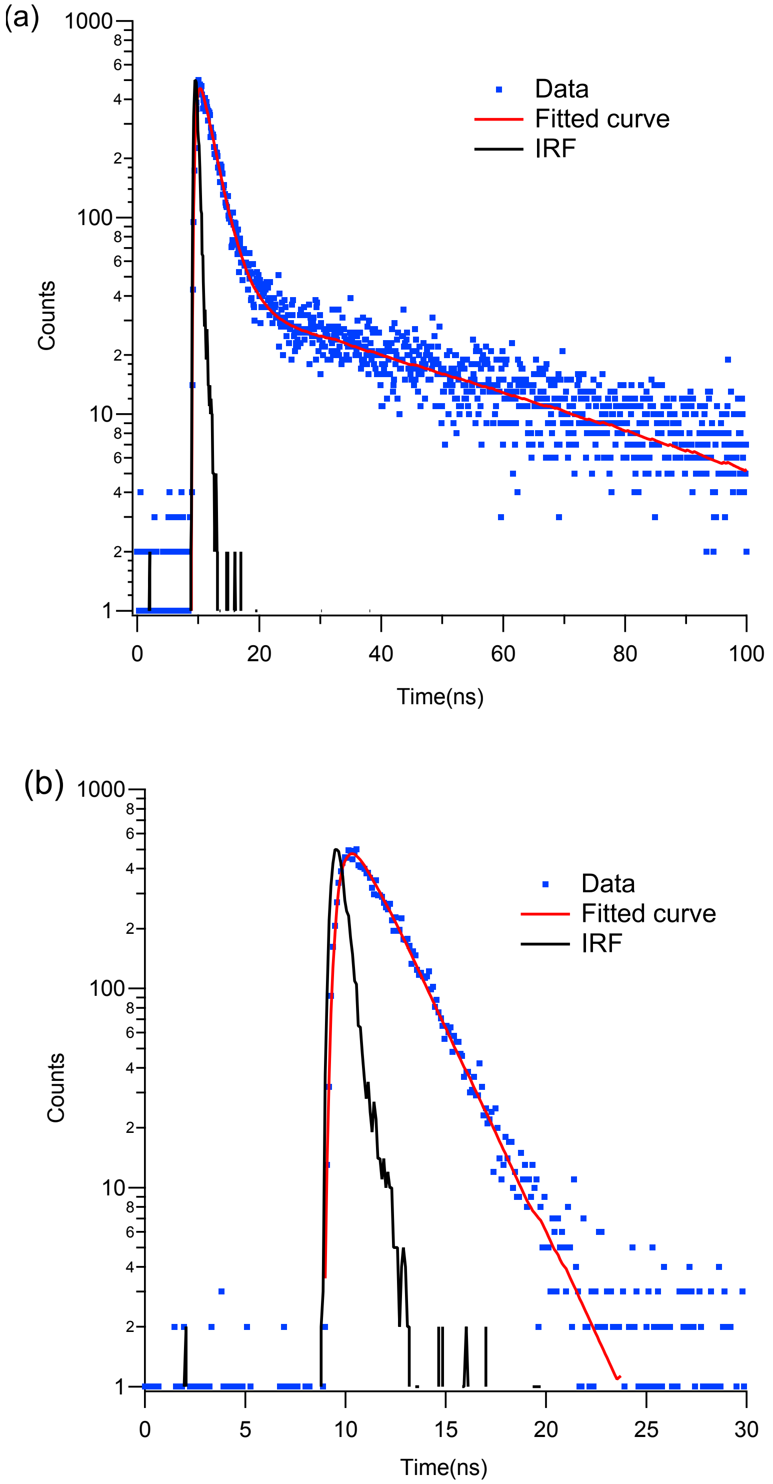

Figure S17. Fluorescence decay analyses of compounds (a) **1** (1.0×10^{-4} mol/L in THF) and (b) **2** (1.0×10^{-3} mol/L in THF). Excitation wavelength: 340 nm, measurement wavelength: 390 nm. The fitting and average lifetime were calculated according to Eqs. (1S)–(2S) below.

$$I(t) = I_0 \exp(-t/\tau) \quad (1S)$$

$$I(t) = A_1\exp(- t/\tau_1) + A_2\exp(- t/\tau_2), \quad (2S)$$

$$<\tau> = (A_1\tau_1^2 + A_2\tau_2^2)/(A_1\tau_1 + A_2\tau_2) \quad (3S)$$

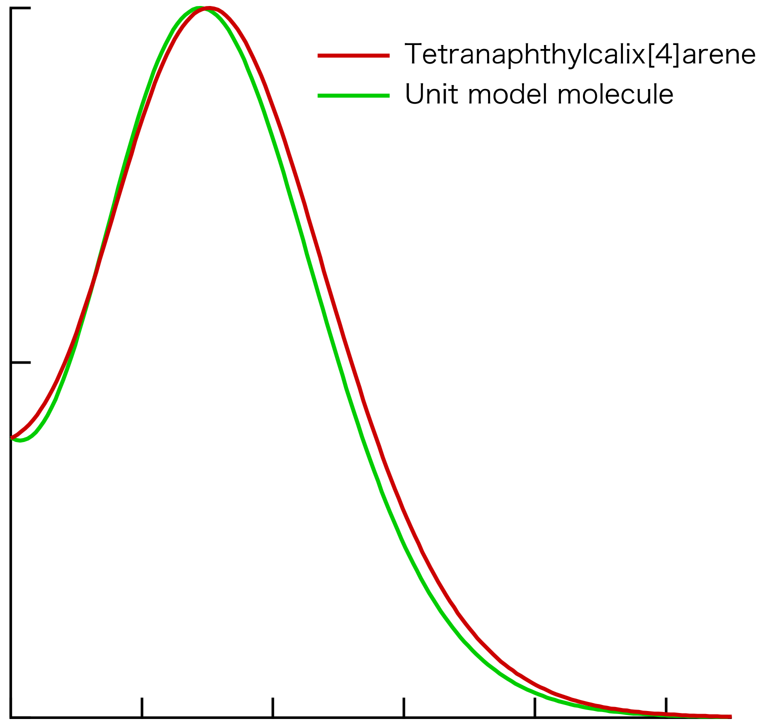

Figure S18. Absorption spectra of tetranaphtylcalix[4]arene and the unit model molecule calculated using TD-DFT. The spectra were normalized at the maximum absorption wavelength in the long wavelength region.

Table S1. Calculated excitation energies and oscillator strengths from the ground state with orbital configuration contributions, for the ground-state optimised geometry of tetranaphtylcalix[4]arene.

Excited state	Transition energy / eV	Oscillator strength f	Configuration and percentage contribution[a]
S_1	4.435 (280 nm)	0.003	HOMO→LUMO+2 (63.7%)
S_2	4.512 (275 nm)	0.109	HOMO→LUMO+3 (53.7%)
S_3	4.568 (271 mm)	0.475	HOMO−1→LUMO+1 (33.7%)
S_4	4.607 (269 nm)	0.370	HOMO−3→LUMO (30.6%)
S_5	4.627 (268 nm)	0.0113	HOMO−6→LUMO+2 (21.0%)

[a]Percentages for the single-particle contributions (y %) to the vertical excited states were calculated using the following expression:

$$y\,\% = \frac{x_i^{\,2}}{\sum_{i=1}^{n} x_i^{\,2}} \times 100$$
,

where x_i is the single-particle transition corresponding to a given vertical excited state.

Table S2. Calculated excitation energies and oscillator strengths from the ground state with orbital configuration contributions, for the ground-state optimised geometry of the unit model molecule.

Excited state	Transition energy / eV	Oscillator strength f	Configuration and percentage contribution[a]
S_1	4.608 (269 nm)	0.2888	HOMO→LUMO (97.6%)
S_2	4.680 (265 nm)	0.0003	HOMO→LUMO+1 (48.7%)
S_3	5.264 (236 mm)	0.0013	HOMO→LUMO+3 (23.9%)
S_4	5.549 (223 nm)	0.1714	HOMO-1→LUMO (52.8%)
S_5	5.809 (213 nm)	0.0128	HOMO→LUMO+2 (53.1%)

[a]The percentages were calculated in the same way as in Table S1.

(a) **(b)**

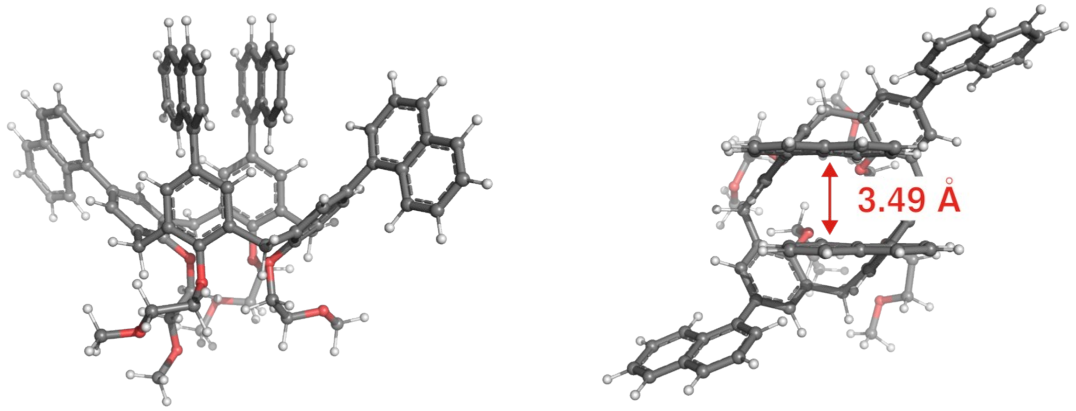

Ground state optimized structure of Tetranaphthylcalix[4]arene

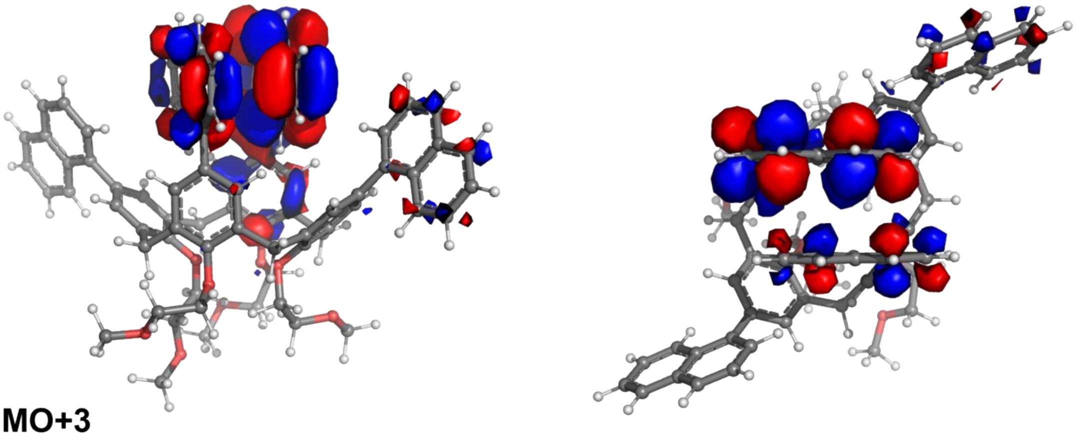

LUMO+3

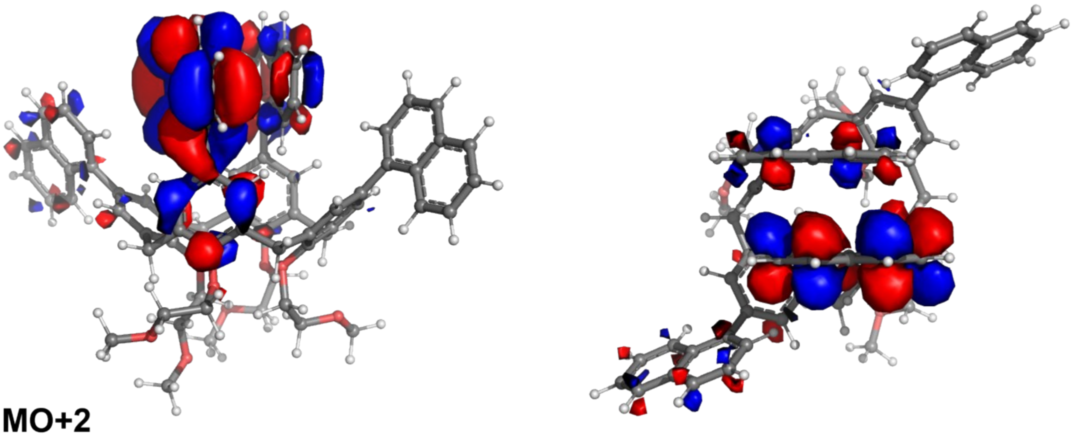

LUMO+2

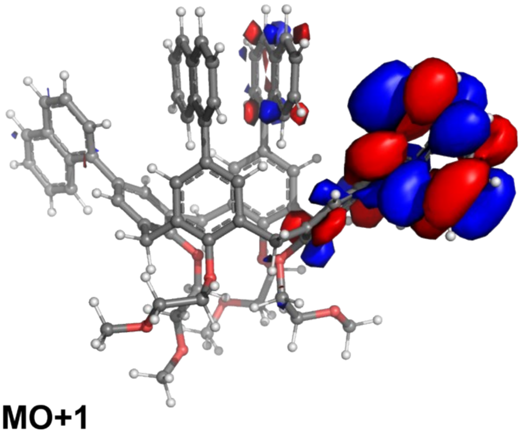

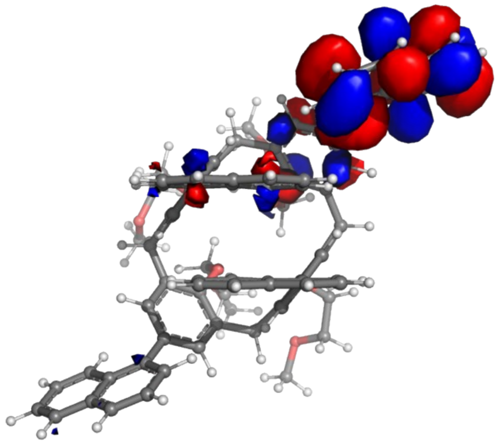

LUMO+1

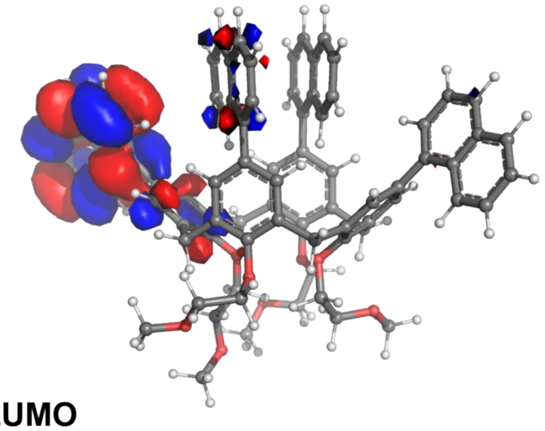

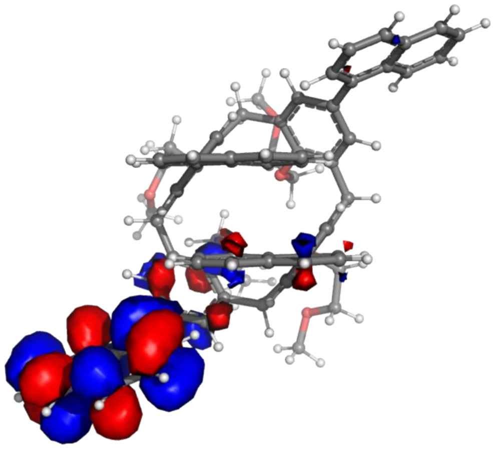

LUMO

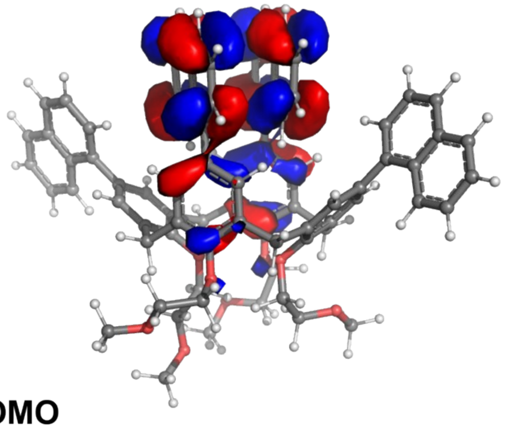

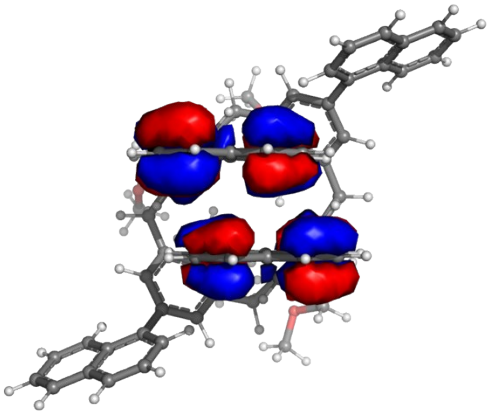

HOMO

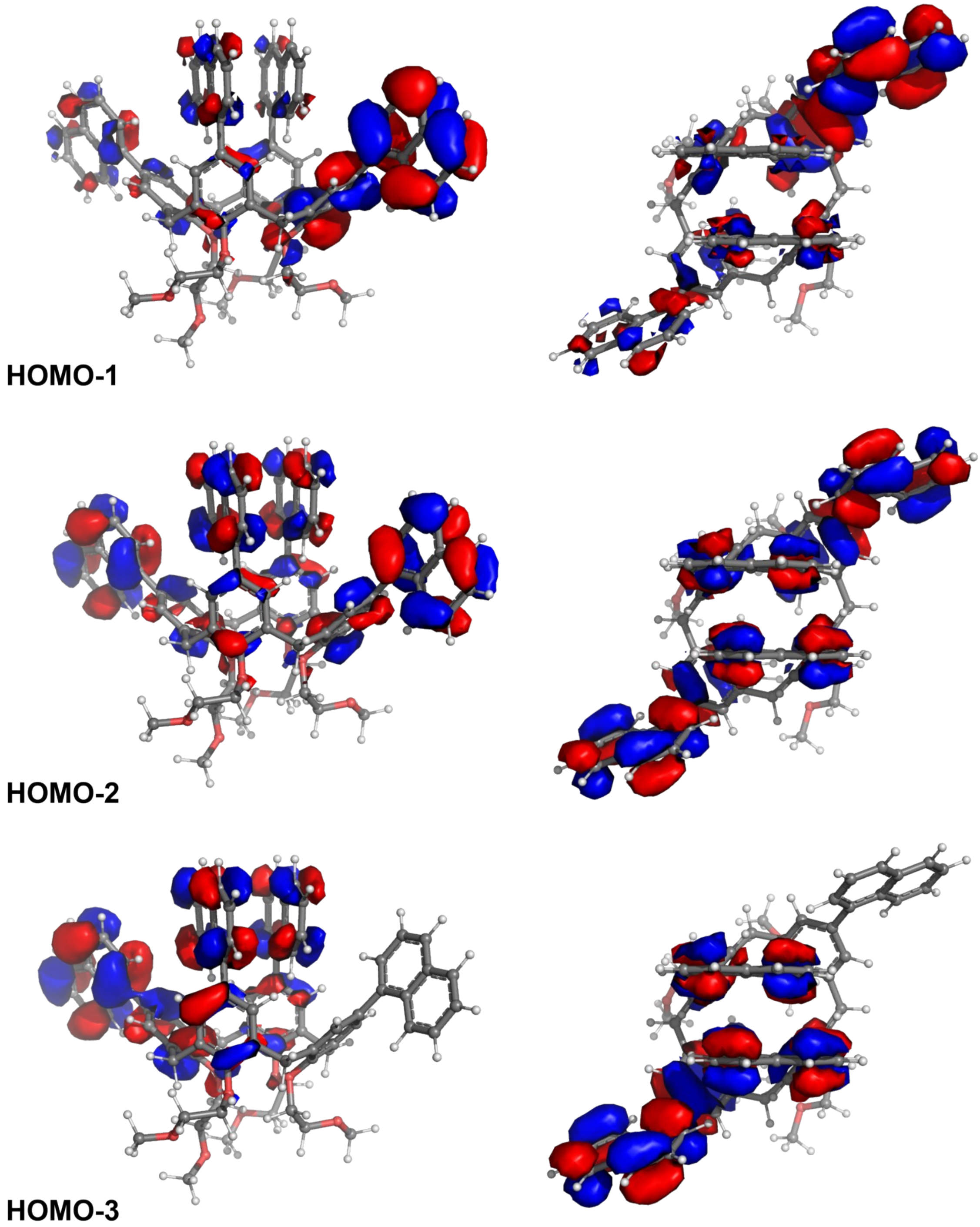

Figure S19. (a) Side and (b) top views of the frontier molecular orbitals of tetranaphthylcalix[4]arene from HOMO-3 to LUMO+3 in the optimised ground state geometry (shown in top row).

(a) (b)

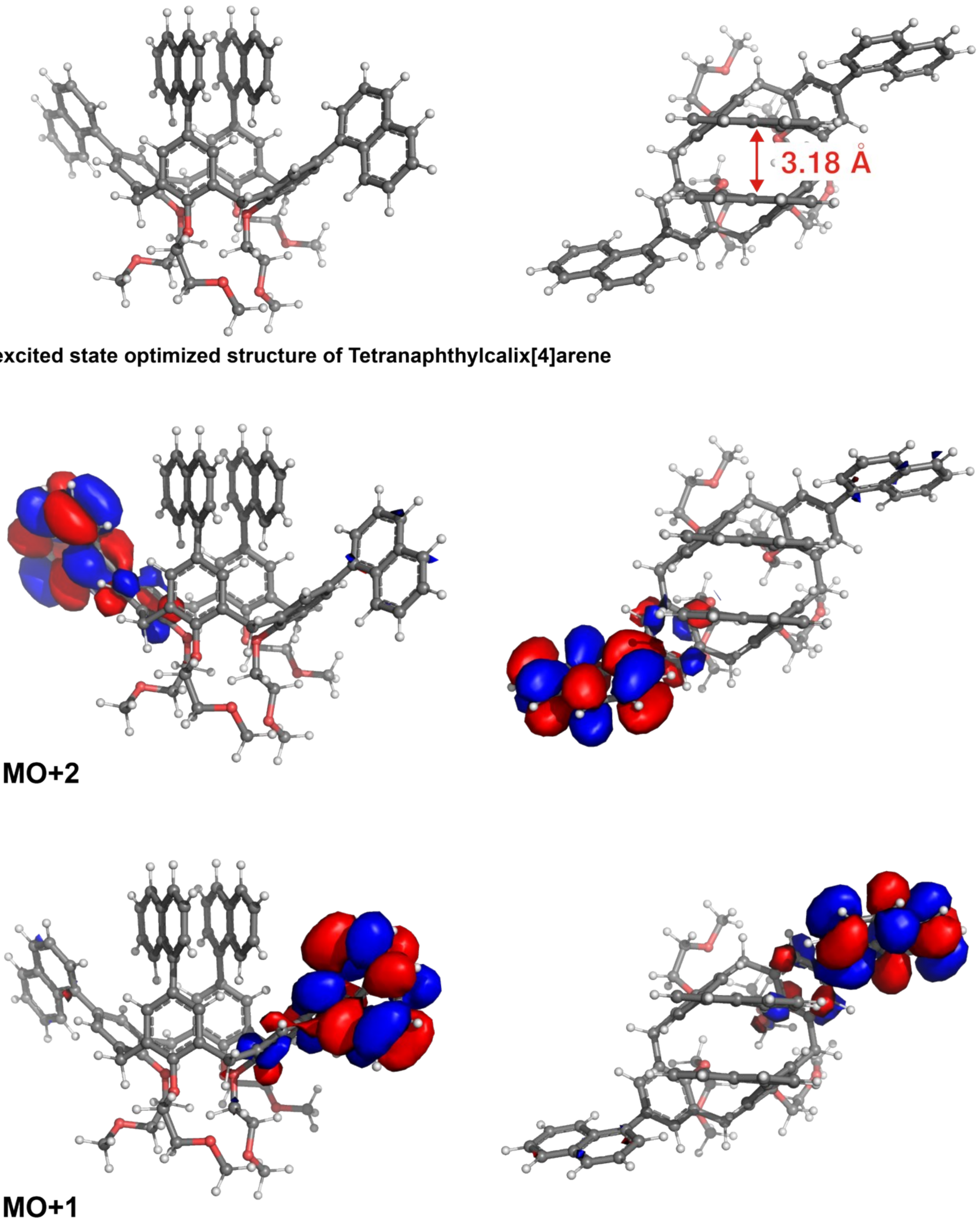

S_1 excited state optimized structure of Tetranaphthylcalix[4]arene

LUMO+2

LUMO+1

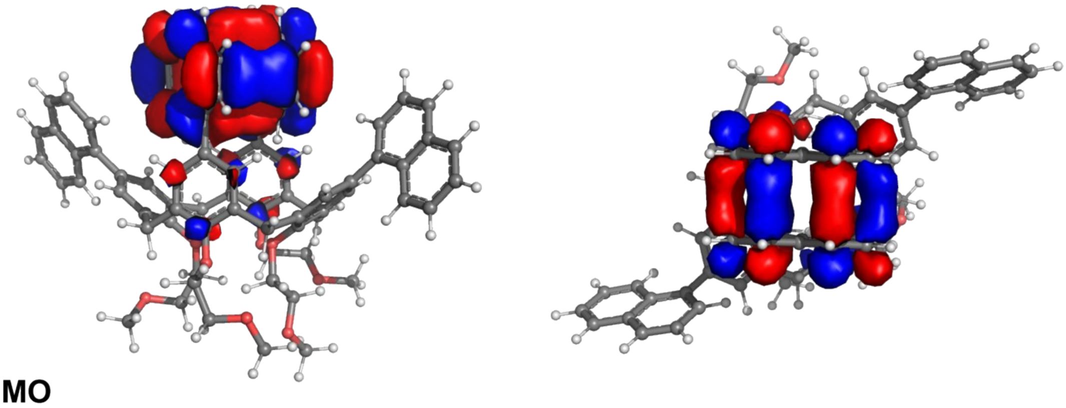

LUMO

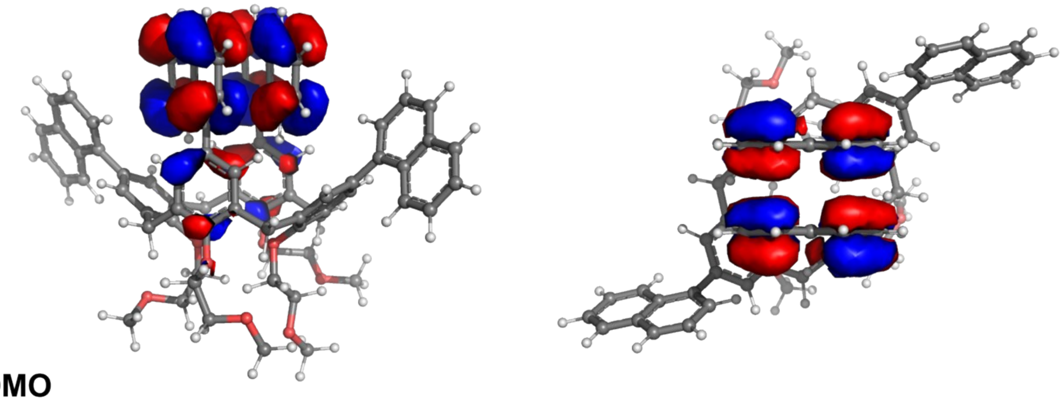

HOMO

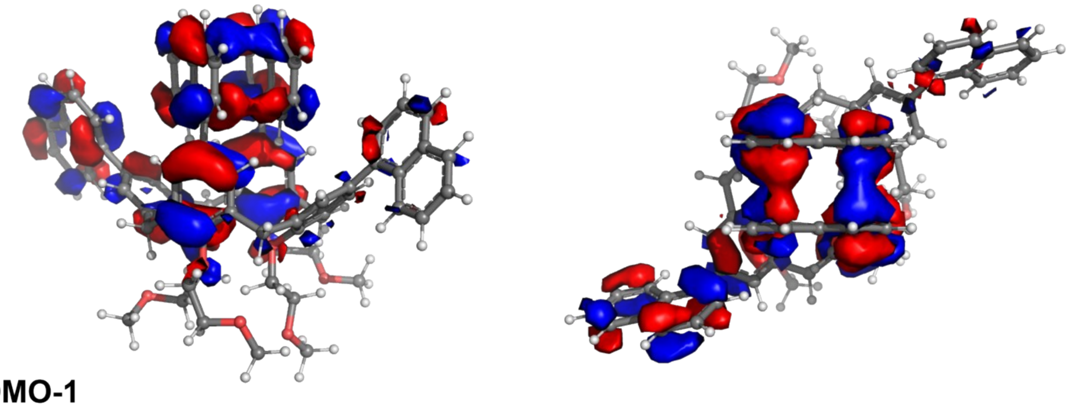

HOMO-1

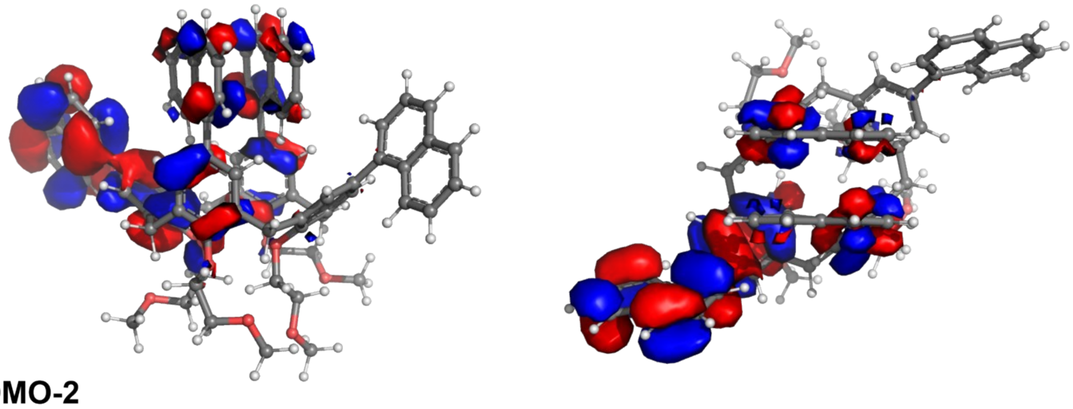

HOMO-2

Figure S20. (a) Side and (b) top views of the frontier molecular orbital for tetranaphthylcalix[4]arene from HOMO-2 to LUMO+2 in the optimised S_1 state geometry (shown in the top row).

Table S3. Crystal data and structure refinement for tetranaphthylcalix[4]arene.

Chemical formula	$C_{88}H_{88}O_{12}$
Formula weight	1337.58 g/mol
Temperature	90 K
Crystal system	Monoclinic
Space group	$P\ 1\ 2_1/c\ 1$
a	13.8913(19) Å
b	29.698(4) Å
c	17.353(2) Å
α	90°
β	104.522(2)°
γ	90°
Volume	6930.2(17) Å^3
Z	4
Reflection	29911
Unique reflection	10611
Final R indices [$I > 2\sigma(I)$], $R1$, w$R2$	$R1 = 0.0731$, w$R2 = 0.1970$
R indices (all data), $R1$, w$R2$	$R1 = 0.1188$, w$R2 = 0.2251$

Table S4. Structure of Tetranaphtylcalix[4]arene, S_0 [DFT-D3/CAM-B3LYP/6-31G (d), E_{sp} = -3691.84119311 hartree].

atom	x[Å]	y[Å]	z[Å]
C	-6.09154	-0.5873	-0.48288
C	-5.93597	-1.78692	-1.13912
H	-4.9683	-2.03423	-1.56259
C	-8.413	-1.223	0.02591
C	-8.19676	-2.45226	-0.64601
H	-9.01007	-3.17113	-0.6947
C	-6.98784	-2.72323	-1.22378
H	-6.81992	-3.66534	-1.73626
C	-7.35776	-0.26979	0.10566
C	-9.88133	0.2793	1.23911
H	-10.84711	0.50026	1.68317
C	-8.84562	1.23776	1.29614
H	-9.02537	2.19529	1.7754
C	-7.62089	0.97125	0.74403
H	-6.8373	1.71894	0.77988
C	-3.10792	1.532	-1.47118
C	-9.66721	-0.91844	0.61355
H	-10.46105	-1.65797	0.55149
C	-4.92638	0.33017	-0.38636
C	-4.24988	0.73921	-1.53511
H	-4.62207	0.42176	-2.50532
C	-3.27395	1.50588	0.95824
C	-4.41828	0.72137	0.85299
H	-4.90675	0.37248	1.75896
C	-2.76245	5.61311	-2.84694
H	-3.7534	5.51295	-3.29443
H	-2.01142	5.23502	-3.55896
H	-2.55739	6.67941	-2.66497
C	-1.53097	4.94637	-0.96934
H	-1.33773	5.97967	-0.63409
H	-0.7078	4.62994	-1.62118
C	-1.57997	4.03127	0.2297
H	-0.73082	4.22752	0.88629

H	-2.50901	4.21835	0.78001
C	-2.64458	1.93426	-0.21596
C	5.56539	-1.95947	0.44087
O	1.75432	2.1886	0.07417
O	0.44548	2.76531	-2.71292
C	-0.45892	-2.81744	-2.56871
C	-0.50649	-2.77144	2.51644
O	-0.00703	2.86631	2.66148
O	-1.4836	2.66024	-0.14409
C	-2.64102	1.76198	2.30915
H	-3.37001	1.51771	3.08939
H	-2.38752	2.81454	2.42816
C	5.13556	-3.09206	1.09382
H	4.13173	-3.11394	1.50534
O	2.68387	5.66314	0.51011
C	0.24799	-3.63411	-3.41994
H	0.958	-3.18518	-4.10797
O	2.7246	4.37237	-3.42988
C	-1.39427	-3.38785	3.3667
H	-1.97692	-2.77699	4.04974
O	1.98494	4.25766	4.2058
O	-2.76233	4.87383	-1.65417
C	5.9496	-4.24031	1.19106
H	5.56907	-5.11958	1.70146
C	7.19571	-4.24844	0.62892
H	7.82671	-5.131	0.68833
C	-1.54337	-4.79009	3.39095
H	-2.25035	-5.24139	4.08037
C	0.05172	-5.03069	-3.43876
H	0.62413	-5.6422	-4.1296
C	7.68956	-3.10157	-0.04207
C	-0.78869	-5.57155	2.56211
H	-0.8866	-6.65357	2.57742
C	-0.86617	-5.60324	-2.6039
H	-1.03474	-6.6766	-2.61579
C	6.87556	-1.93675	-0.13562

C	0.13115	-4.98512	1.65735
C	-1.61115	-4.80792	-1.69776
C	7.41425	-0.79011	-0.77723
H	6.8159	0.11204	-0.82791
C	0.27259	-3.56867	1.61422
C	-1.40318	-3.39949	-1.65989
C	8.67264	-0.80724	-1.3175
H	9.06517	0.08197	-1.80127
C	1.1578	-3.01467	0.64991
H	1.235	-1.93929	0.54666
C	-2.12129	-2.64378	-0.69384
H	-1.93555	-1.581	-0.59833
C	9.46812	-1.97195	-1.24412
H	10.46297	-1.9749	-1.6788
C	1.8932	-3.81894	-0.17982
H	2.56677	-3.36972	-0.90206
C	-3.0248	-3.24228	0.14366
H	-3.56531	-2.64043	0.86665
C	8.98605	-3.08799	-0.61652
H	9.59398	-3.98579	-0.54371
C	1.77489	-5.22318	-0.11025
H	2.35776	-5.84729	-0.77987
C	-3.25344	-4.633	0.07864
H	-3.9681	-5.09406	0.75273
C	3.13295	0.781	1.40429
C	0.90473	-5.78764	0.78144
H	0.78411	-6.86668	0.82723
C	-2.55279	-5.39502	-0.81551
H	-2.70045	-6.47085	-0.85865
C	4.07667	-0.23998	1.47982
H	4.37403	-0.61945	2.45365
C	-1.395	0.91508	2.49681
C	1.3076	0.53669	-2.55598
C	4.63824	-0.80272	0.33542
C	-1.49847	-0.47165	2.45594
H	-2.47501	-0.92292	2.30627

C	1.06631	-0.83261	-2.51372
H	1.90289	-1.51061	-2.37064
C	4.22474	-0.32219	-0.908
H	4.6243	-0.77954	-1.80917
C	-0.37694	-1.29373	2.55304
C	-0.22344	-1.35272	-2.60858
C	3.27527	0.68797	-1.02771
C	0.86915	-0.68638	2.67647
H	1.75807	-1.30634	2.73199
C	-1.28236	-0.45769	-2.73497
H	-2.29588	-0.84108	-2.79257
C	2.75118	1.25043	0.14373
C	1.01965	0.69471	2.68873
C	-1.08725	0.91812	-2.74821
C	2.12572	3.51416	-0.28807
H	1.24914	3.95544	-0.7571
H	2.93177	3.5125	-1.02493
C	-0.12699	1.48913	2.63266
C	0.21924	1.40339	-2.68552
C	2.52109	4.32285	0.92967
H	1.7444	4.24392	1.69676
H	3.45963	3.93776	1.36184
C	-0.23368	3.47846	3.92308
H	-1.0688	2.99243	4.44334
H	-0.50837	4.51523	3.70869
C	0.6791	3.31432	-4.00012
H	-0.27474	3.47018	-4.52502
H	1.29644	2.63633	-4.60163
C	3.05619	6.50282	1.57425
H	3.07489	7.52782	1.19658
H	4.05752	6.2472	1.9564
H	2.3456	6.43174	2.41025
C	0.99139	3.46385	4.81053
H	0.72801	3.86062	5.80485
H	1.34397	2.43047	4.94513
C	1.40054	4.63019	-3.82688

H	1.38337	5.19048	-4.77613
H	0.8771	5.23652	-3.07287
C	2.72271	1.0562	-2.38824
H	3.36423	0.60507	-3.1529
H	2.74391	2.13237	-2.55516
C	3.22232	4.19792	4.87189
H	3.91301	4.84404	4.32642
H	3.62296	3.1739	4.88185
H	3.14154	4.5533	5.90986
C	-2.29469	1.83423	-2.70931
H	-1.97812	2.87429	-2.70255
H	-2.91744	1.67466	-3.59633
C	2.4213	1.27272	2.64617
H	2.37195	2.35913	2.67687
H	2.98406	0.94252	3.5271
C	3.39151	5.52001	-2.95092
H	4.42926	5.23275	-2.76927
H	2.95488	5.87513	-2.00752
H	3.37325	6.3324	-3.69263

Table S5. Structure of Tetranaphtylcalix[4]arene, S_1 [DFT-D3/CAM-B3LYP/6-31G (d), E_{sp} = -3691.81643238 hartree].

atom	x[Å]	y[Å]	z[Å]
C	-6.21594	-0.605	-0.52636
C	-6.10234	-1.79038	-1.21673
H	-5.14638	-2.05374	-1.65706
C	-8.55184	-1.18595	-0.0158
C	-8.37728	-2.40264	-0.72177
H	-9.21165	-3.09597	-0.78227
C	-7.18193	-2.69268	-1.318
H	-7.04655	-3.62464	-1.85788
C	-7.46873	-0.26588	0.07969
C	-9.96811	0.32613	1.24611
H	-10.9239	0.56305	1.7033
C	-8.90548	1.25354	1.31743

H	-9.05471	2.20414	1.82036
C	-7.69272	0.96635	0.74948
H	-6.88904	1.6916	0.79654
C	-3.1704	1.43826	-1.48243
C	-9.79249	-0.86116	0.58951
H	-10.60737	-1.57626	0.5153
C	-5.02384	0.27538	-0.41503
C	-4.3289	0.67184	-1.55803
H	-4.69932	0.36479	-2.53223
C	-3.34891	1.40061	0.9457
C	-4.50917	0.64072	0.83012
H	-5.00515	0.2909	1.73163
C	-2.82932	5.49714	-2.83345
H	-3.82033	5.39398	-3.28021
H	-2.07774	5.12088	-3.54594
H	-2.62728	6.56417	-2.65218
C	-1.59415	4.8355	-0.95678
H	-1.40485	5.86853	-0.61859
H	-0.77117	4.52511	-1.61158
C	-1.63793	3.91673	0.23972
H	-0.78651	4.10953	0.89399
H	-2.56402	4.10146	0.7957
C	-2.71222	1.83185	-0.22286
C	5.76638	-1.75728	0.45755
O	1.71139	2.15215	0.08008
O	0.38589	2.7046	-2.74644
C	-0.37982	-2.86401	-2.15221
C	-0.45945	-2.84809	2.09018
O	-0.09277	2.76519	2.71221
O	-1.54532	2.54656	-0.14067
C	-2.69878	1.62191	2.29427
H	-3.40861	1.33613	3.07818
H	-2.46235	2.67466	2.44681
C	5.42957	-2.90735	1.13461
H	4.43659	-2.99446	1.56333
O	2.44447	5.66615	0.54953

C	0.47063	-3.74273	-2.85384
H	1.23761	-3.31956	-3.49353
O	2.61279	4.37648	-3.42333
C	-1.46739	-3.53632	2.79635
H	-2.13565	-2.96558	3.43237
O	1.85256	4.1744	4.26576
O	-2.82611	4.75836	-1.64047
C	6.32517	-3.99254	1.23705
H	6.01612	-4.88838	1.76648
C	7.56022	-3.92064	0.65562
H	8.25357	-4.75486	0.71801
C	-1.59004	-4.90954	2.72747
H	-2.37543	-5.41377	3.28146
C	0.31933	-5.11264	-2.77562
H	0.98861	-5.76591	-3.32621
C	7.96119	-2.75166	-0.03866
C	-0.71078	-5.65012	1.93675
H	-0.80106	-6.73107	1.8837
C	-0.68819	-5.65919	-1.98043
H	-0.81283	-6.73624	-1.91941
C	7.06486	-1.64916	-0.13669
C	0.34259	-5.02186	1.24389
C	-1.59362	-4.83045	-1.29031
C	7.51422	-0.4758	-0.79867
H	6.85449	0.3822	-0.85154
C	0.46286	-3.59145	1.28517
C	-1.42761	-3.40535	-1.34068
C	8.76402	-0.41132	-1.3551
H	9.087	0.49738	-1.85384
C	1.44179	-2.99816	0.45914
H	1.4903	-1.92052	0.37154
C	-2.26584	-2.62433	-0.51597
H	-2.09992	-1.55788	-0.43633
C	9.64039	-1.51614	-1.27887
H	10.62743	-1.45481	-1.7268
C	2.36619	-3.76884	-0.25329

H	3.14299	-3.26738	-0.8215
C	-3.32207	-3.1927	0.20302
H	-3.98257	-2.54387	0.76877
C	9.24606	-2.65469	-0.63088
H	9.91672	-3.50633	-0.55456
C	2.28685	-5.15186	-0.238
H	2.99898	-5.75064	-0.79645
C	-3.5195	-4.56386	0.19618
H	-4.33458	-5.00558	0.75994
C	3.14358	0.80344	1.41504
C	1.2501	-5.76849	0.46109
H	1.15777	-6.85076	0.45561
C	-2.6282	-5.37826	-0.50083
H	-2.75184	-6.45723	-0.48803
C	4.14998	-0.15519	1.49201
H	4.46168	-0.52258	2.46593
C	-1.43176	0.79399	2.42575
C	1.30503	0.50593	-2.48628
C	4.75729	-0.67166	0.34822
C	-1.49984	-0.58327	2.26944
H	-2.46479	-1.03851	2.06929
C	1.09388	-0.85729	-2.33576
H	1.9479	-1.49887	-2.14115
C	4.31496	-0.21946	-0.89674
H	4.74168	-0.65184	-1.79777
C	-0.3558	-1.38984	2.27742
C	-0.18968	-1.41514	-2.34382
C	3.30488	0.7302	-1.01608
C	0.87285	-0.75767	2.47408
H	1.77771	-1.35449	2.49772
C	-1.26692	-0.5495	-2.541
H	-2.27272	-0.95322	-2.56914
C	2.75028	1.26167	0.15471
C	0.98651	0.61998	2.61207
C	-1.09958	0.82342	-2.67247
C	2.0318	3.49361	-0.27329

H	1.15049	3.8964	-0.76706
H	2.85718	3.52773	-0.98814
C	-0.17763	1.38961	2.60661
C	0.19526	1.34115	-2.65554
C	2.35741	4.31518	0.9568
H	1.57519	4.18178	1.71114
H	3.31037	3.98384	1.40175
C	-0.33527	3.31234	3.99987
H	-1.13637	2.76199	4.50918
H	-0.66612	4.34145	3.83269
C	0.64352	3.21021	-4.0463
H	-0.29589	3.3049	-4.61021
H	1.31067	2.53662	-4.5975
C	2.75789	6.51679	1.62398
H	2.71661	7.54457	1.25579
H	3.77019	6.31877	2.01125
H	2.04756	6.39476	2.45434
C	0.90389	3.325	4.86724
H	0.6425	3.68478	5.87608
H	1.30086	2.30399	4.9682
C	1.30096	4.56183	-3.89486
H	1.30785	5.08432	-4.86537
H	0.71474	5.17197	-3.19174
C	2.71237	1.06261	-2.36831
H	3.34497	0.61559	-3.14295
H	2.70285	2.1369	-2.5504
C	3.10126	4.1543	4.91288
H	3.7556	4.84049	4.37149
H	3.54547	3.14846	4.89564
H	3.02115	4.48452	5.95919
C	-2.32669	1.7148	-2.705
H	-2.0282	2.76061	-2.72555
H	-2.91773	1.51274	-3.60512
C	2.37486	1.23279	2.6449
H	2.29571	2.31645	2.71062
H	2.91322	0.88689	3.5352

C	3.19477	5.55992	-2.92178
H	4.23562	5.32826	-2.68647
H	2.69485	5.89692	-2.00339
H	3.17152	6.36649	-3.66954

Table S6. Structure of Unit model molecule, S_0 [DFT-D3/CAM-B3LYP/6-31G (d), E_{sp} = -963.428287148 hartree].

atom	x[Å]	y[Å]	z[Å]
C	-1.07063	0.23233	1.25427
C	0.31279	0.17619	1.13192
C	0.93171	-0.70244	0.23917
C	0.13026	-1.54621	-0.5276
C	-1.25958	-1.51959	-0.43573
C	-1.84005	-0.61707	0.45522
C	2.41314	-0.7921	0.14865
C	3.22455	0.35091	-0.14769
C	4.6408	0.20166	-0.17548
C	5.21645	-1.07052	0.06665
C	4.42231	-2.1544	0.31706
C	3.01983	-2.00883	0.35935
C	2.67977	1.62733	-0.45005
C	3.49013	2.69561	-0.72878
C	4.89479	2.55035	-0.72546
C	5.45195	1.32955	-0.46021
O	-3.21265	-0.58936	0.60241
C	-3.8884	0.25489	-0.31783
C	-5.35502	0.22531	0.05346
O	-6.03416	1.04964	-0.85858
C	-7.41682	1.10536	-0.61605
C	-1.74441	1.15709	2.23099
C	-2.11712	-2.43459	-1.26728
H	0.92733	0.81785	1.75679
H	0.60271	-2.23886	-1.21866

H	6.29829	-1.16767	0.04347
H	4.86385	-3.1294	0.49831
H	2.40058	-2.87058	0.58866
H	1.60329	1.74785	-0.47081
H	3.05033	3.6607	-0.9609
H	5.52708	3.40537	-0.94382
H	6.531	1.20176	-0.46961
H	-3.75665	-0.09862	-1.34755
H	-3.50741	1.28197	-0.26035
H	-5.4812	0.58034	1.08835
H	-5.7253	-0.81117	0.01179
H	-7.85353	1.76413	-1.36937
H	-7.63741	1.50977	0.38357
H	-7.88315	0.11164	-0.69662
H	-1.0109	1.64779	2.87541
H	-2.45343	0.61101	2.85926
H	-2.31411	1.93906	1.71697
H	-1.57134	-3.3418	-1.53939
H	-2.43223	-1.95107	-2.19929
H	-3.02196	-2.71735	-0.72428

6. Supplementary References

1. Gaussian 16 Rev. C.01, M. J. Frisch, G. W. Trucks, H. B. Schlegel, G. E. Scuseria, M. A. Robb, J. R. Cheeseman, G. Scalmani, V. Barone, G. A. Petersson, H. Nakatsuji, X. Li, M. Caricato, A. V. Marenich, J. Bloino, B. G. Janesko, R. Gomperts, B. Mennucci, H. P. Hratchian, J. V. Ortiz, A. F. Izmaylov, J. L. Sonnenberg, Williams, F. Ding, F. Lipparini, F. Egidi, J. Goings, B. Peng, A. Petrone, T. Henderson, D. Ranasinghe, V. G. Zakrzewski, J. Gao, N. Rega, G. Zheng, W. Liang, M. Hada, M. Ehara, K. Toyota, R. Fukuda, J. Hasegawa, M. Ishida, T. Nakajima, Y. Honda, O. Kitao, H. Nakai, T. Vreven, K. Throssell, J. A. Montgomery Jr., J. E. Peralta, F. Ogliaro, M. J. Bearpark, J. J. Heyd, E. N. Brothers, K. N. Kudin, V. N. Staroverov, T. A. Keith, R. Kobayashi, J. Normand, K. Raghavachari, A. P. Rendell, J. C. Burant, S. S. Iyengar, J. Tomasi, M. Cossi, J. M. Millam, M. Klene, C. Adamo, R. Cammi, J. W. Ochterski, R. L. Martin, K. Morokuma, O. Farkas, J. B. Foresman and D. J. Fox, Gaussian, Inc., Wallingford, CT, 2016.
2. T. Yanai, D. P. Tew and N. C. Handy, *Chem. Phys. Lett.*, 2004, **393**, 51-57.
3. P. C. Hariharan and J. A. Pople, *Theo. Chim. Acta*, 1973, **28**, 213-222.
4. S. Grimme, *J. Comput. Chem.*, 2004, **25**, 1463-1473.
5. P. K. Lo, D. Chen, Q. Meng and M. S. Wong, *Chem. Mater.*, 2006, **18**, 3924-3930.
6. K. E. Brown, B. S. Veldkamp, D. T. Co and M. R. Wasielewski, *J. Phys. Chem. Lett.*, 2012, **3**, 2362-2366.
7. X. H. Sun, W. Li, P. F. Xia, H.-B. Luo, Y. Wei, M. S. Wong, Y.-K. Cheng and S. Shuang, *J. Org. Chem.*, 2007, **72**, 2419-2426.
